# Complex and extensive post-transcriptional regulation revealed by integrative proteomic and transcriptomic analysis of metabolite stress response in *Clostridium acetobutylicum*

**DOI:** 10.1186/s13068-015-0260-9

**Published:** 2015-06-10

**Authors:** Keerthi P. Venkataramanan, Lie Min, Shuyu Hou, Shawn W. Jones, Matthew T. Ralston, Kelvin H. Lee, E. Terry Papoutsakis

**Affiliations:** 15 Innovation Way, Delaware Biotechnology Institute, University of Delaware, Newark, DE 19711 USA; 150 Academy Street, Department of Chemical and Biomolecular Engineering, University of Delaware, Newark, DE 19711 USA; 15 Innovation Way, Center for Bioinformatics and Computational Biology, University of Delaware, Newark, DE 19711 USA

**Keywords:** Metabolite stress, iTRAQ proteome, Microarray, RNAseq, Integrative analysis, Post-transcriptional regulation

## Abstract

**Background:**

*Clostridium acetobutylicum* is a model organism for both clostridial biology and solvent production. The organism is exposed to its own toxic metabolites butyrate and butanol, which trigger an adaptive stress response. Integrative analysis of proteomic and RNAseq data may provide novel insights into post-transcriptional regulation.

**Results:**

The identified iTRAQ-based quantitative stress proteome is made up of 616 proteins with a 15 % genome coverage. The differentially expressed proteome correlated poorly with the corresponding differential RNAseq transcriptome. Up to 31 % of the differentially expressed proteins under stress displayed patterns opposite to those of the transcriptome, thus suggesting significant post-transcriptional regulation. The differential proteome of the translation machinery suggests that cells employ a different subset of ribosomal proteins under stress. Several highly upregulated proteins but with low mRNA levels possessed mRNAs with long 5′UTRs and strong RBS scores, thus supporting the argument that regulatory elements on the long 5′UTRs control their translation. For example, the oxidative stress response rubrerythrin was upregulated only at the protein level up to 40-fold without significant mRNA changes. We also identified many leaderless transcripts, several displaying different transcriptional start sites, thus suggesting mRNA-trimming mechanisms under stress. Downregulation of Rho and partner proteins pointed to changes in transcriptional elongation and termination under stress.

**Conclusions:**

The integrative proteomic-transcriptomic analysis demonstrated complex expression patterns of a large fraction of the proteome. Such patterns could not have been detected with one or the other omic analyses. Our analysis proposes the involvement of specific molecular mechanisms of post-transcriptional regulation to explain the observed complex stress response.

**Electronic supplementary material:**

The online version of this article (doi:10.1186/s13068-015-0260-9) contains supplementary material, which is available to authorized users.

## Background

*Clostridium acetobutylicum* is a model organism for the acetone, butanol, and ethanol (ABE) fermentation and for clostridial biology in general. *C. acetobutylicum* has the ability to ferment a very large range of carbon sources for the production of a wide array of products, including carboxylic acids (butyrate and acetate) and solvents (ABE) [1, 2]. These products are toxic and affect cell growth and survival. A deeper understanding of the response and tolerance to metabolite and more generally chemical toxicity can lead to robust and rational design of strains suitable for industrial bioprocessing [1, 3–5]. More broadly, *Clostridium* organisms are predominantly soil bacteria that, in their natural milieu, are exposed to a large variety of chemicals, many of which are toxic to the cells. As a result, they have evolved to develop specific mechanisms to resist chemical toxicity [6, 7, 4]. Although response to chemical toxicity is not necessarily related to tolerance, several studies have demonstrated that components (genes/proteins, programs) of the well-preserved heat shock protein (HSP or stress) response can be engaged to develop tolerant strains [4, 8–11]. We will show here that regulation of this core HSP response is considerably more complex that has been so far revealed by transcriptional studies. These are discussed next.

Several transcriptomic studies using DNA microarrays and, recently, RNAseq, have dissected the RNome dynamics of this organism. These studies have unveiled the transcriptional program associated with culture-phase-specific metabolism and physiological changes [12, 13]. Transcriptional analyses have also shed light into the dynamics of the organism’s stress response to toxic fermentation products, notably butanol, butyrate, and acetate, in batch [6, 7, 14–16] and continuous cultures [17, 18]. Microarray data have been used to identify and characterize the stress-responsive gene network using phylogenetic RSAT footprinting analyses [16], while the RNAseq studies have been used to identify stress-responsive non-coding small RNAs (sRNAs) that might be part of the regulatory network of the stress response [15].

In view of the complex post-transcriptional events, such as differential mRNA and protein stability, differential regulation of the translation process, and the involvement of regulatory sRNAs, integrative analysis of transcriptomic and proteomic data could provide many novel insights not possible using only one type of omic data [19].

Here, we present the analysis of a large set of proteomic data aiming to examine at the proteome level the response of *C. acetobutylicum* to butanol and butyrate stress and a comparative analysis of these proteomic data against two sets of transcriptomic data, one based on microarray analysis [16] and the second on RNAseq [15]. These extensive omic data were collected from the same master cultures. Their analysis aims to provide a more comprehensive understanding of the metabolite stress response with emphasis on identifying new regulatory mechanisms not accessible through either transcriptomic or proteomic analyses alone.

## Results

### The metabolite stress proteome of *C. acetobutylicum*

#### A large and deep set of proteomic data to characterize the dynamic cellular response to butanol and butyrate stress

*C. acetobutylicum* cultures were grown anaerobically in 4 L bioreactors at 37 °C on defined CGM with 40 g/L glucose. As in the corresponding microarray [16] and RNAseq [15] studies, cultures were stressed with three levels of butyrate (0 mM - control; 30 mM - low; 40 mM - medium; and 50 mM - high) and three levels of butanol (0 mM - control; 30 mM - low; 60 mM - medium; and 90 mM - high) stress at a cell density (A_600_) of 1.0. The effect of butanol stress was dose dependent and had a severe impact on cell growth and glucose utilization in comparison to the non-stressed control cultures (Additional file 1: Figure S1) On the other hand, although butyrate stress affected substrate utilization, its impact on cell growth was not severe, as the growth of the butyrate-stressed cultures was similar to the non-stressed control cultures. Clostridial metabolism includes acid reassimilation leading to solvent formation. It appears that uptake of the exogenous butyrate minimizes the impact of this carboxylic acid on cell growth [12, 15, 16].

**Fig. 1:**
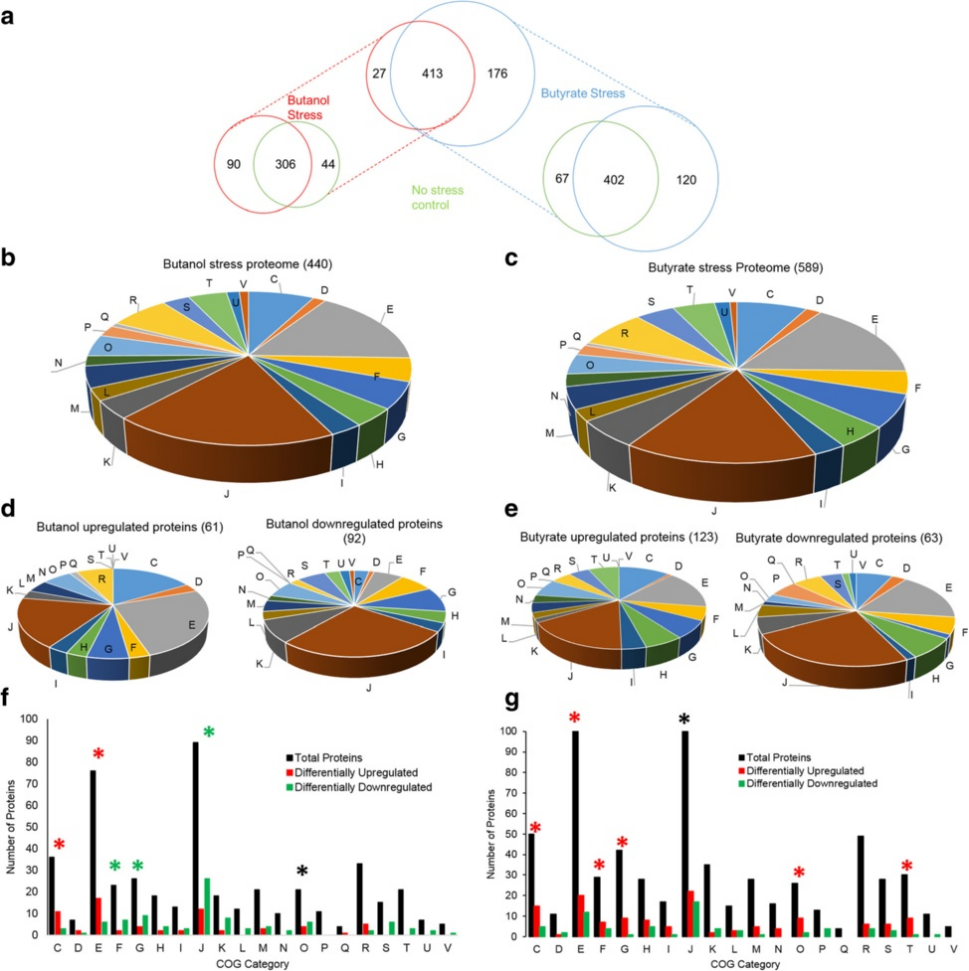
Clostridial proteomic summary under metabolite stress. **a** Comparison of the butanol (red) and butyrate (blue) stress proteome. Comparison of proteome between non-stress control condition and **b** butanol stress. Comparison of proteome between non-stress control condition and **c** butyrate stress. Distribution of the stress proteome into various COG functional groups **d** butanol stress and **e** butyrate stress. Differential expression within COG categories **f** butanol stress and **g** butyrate stress. Red asterisks: COG category enriched with upregulated proteins; green asterisks: COG category enriched in downregulated proteins; black asterisks: COG category equally enriched in up- and downregulated proteins. C: energy production and conversion; D: cell division and chromosome partitioning; E: amino acid transport and metabolism; F: nucleotide transport and metabolism; G: carbohydrate transport and metabolism; H: coenzyme metabolism; I: lipid metabolism; J: translation, ribosomal structure and biogenesis; K: transcription; L: DNA replication, recombination, and repair; M: cell envelope biogenesis, outer membrane; N: cell motility and secretion; O: posttranslational modification, protein turnover, chaperones; P: inorganic ion transport and metabolism; Q: secondary metabolites biosynthesis, transport, and catabolism; R: general function prediction only; S: function unknown; T: signal transduction mechanisms; U: intracellular trafficking, secretion, and vesicular transport; V: defense mechanisms

The stress proteome was identified using iTRAQ (4-plex) samples from 15, 45, and 75 min post stress and a reference pool that was created by pooling equal amounts of proteins from all samples for each metabolite stress (see Materials and methods). A total of 440 and 589 proteins (Fig. 1, panels a, b, and c) were identified under butanol and butyrate stress, respectively. Four hundred thirteen proteins were detected under both butanol and butyrate stress (Fig. 1). Stressed samples were compared against the non-stress sample to identify proteins that were expressed only during stress and proteins that were expressed only during non-stress control condition. Comparing the proteome of butanol-stressed sample with its corresponding non-stressed control, 90 proteins were exclusively detected only under butanol stress conditions with no expression under non-stress control, while 44 proteins were exclusively detected only under the corresponding control, non-stress conditions with no expression under butanol stress (Figs. 1 and 2 and Additional file 1: Figure S2). Similarly, between butyrate-stressed and non-stress control samples, 120 and 67 proteins were exclusively detected only under butyrate-stress versus the corresponding control, non-stress conditions, respectively (Figs. 1 and 3 Additional file 1: Figure S2). Proteins expressed only under either stress or control conditions likely have an important role in these metabolite stress responses.

**Fig. 2:**
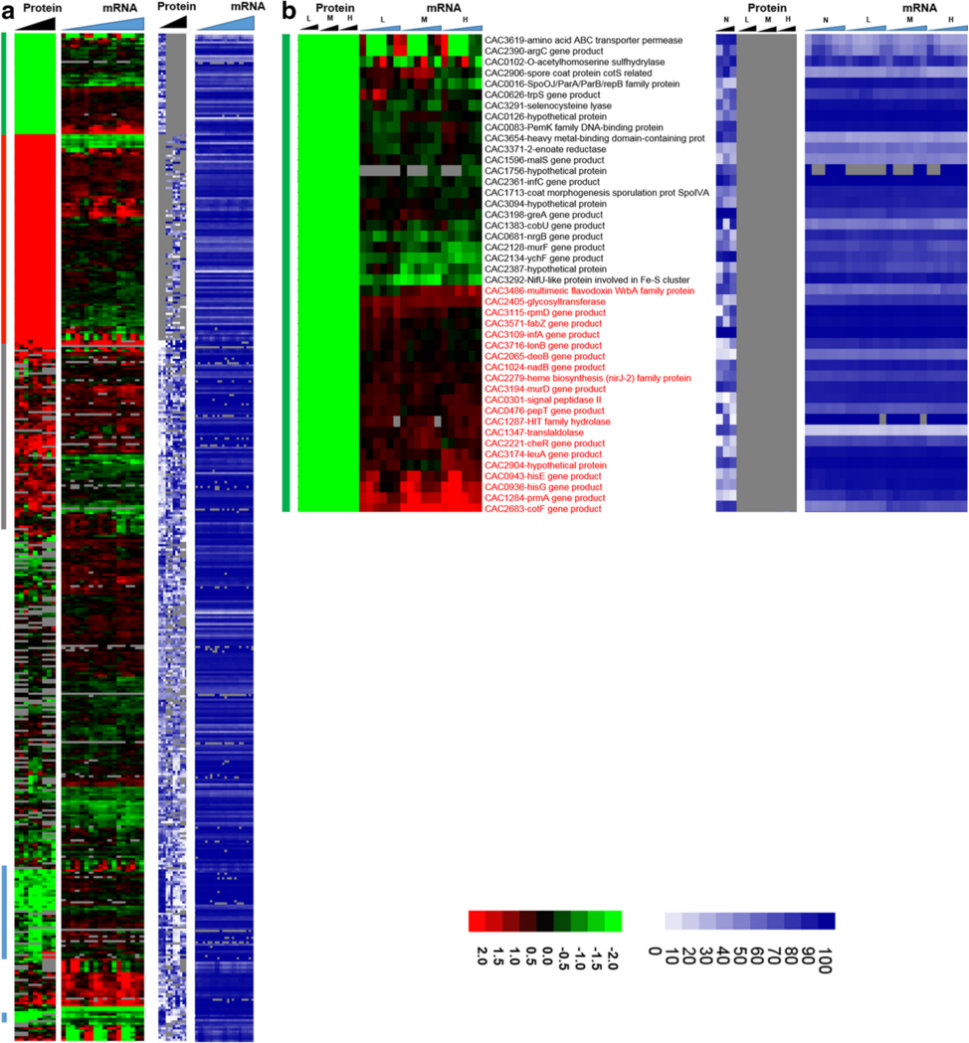
Heat map of proteomic and transcriptomic (microarrays) differential expression between butanol stress and control condition. Differential gene expression of stress versus non-stress control is displayed in red-green and protein and mRNA abundance percentile ranking is shown in blue plots. **a** Butanol stress proteome **b** proteins expressed under non-stress control condition only (green vertical bar).

**Fig. 2:**
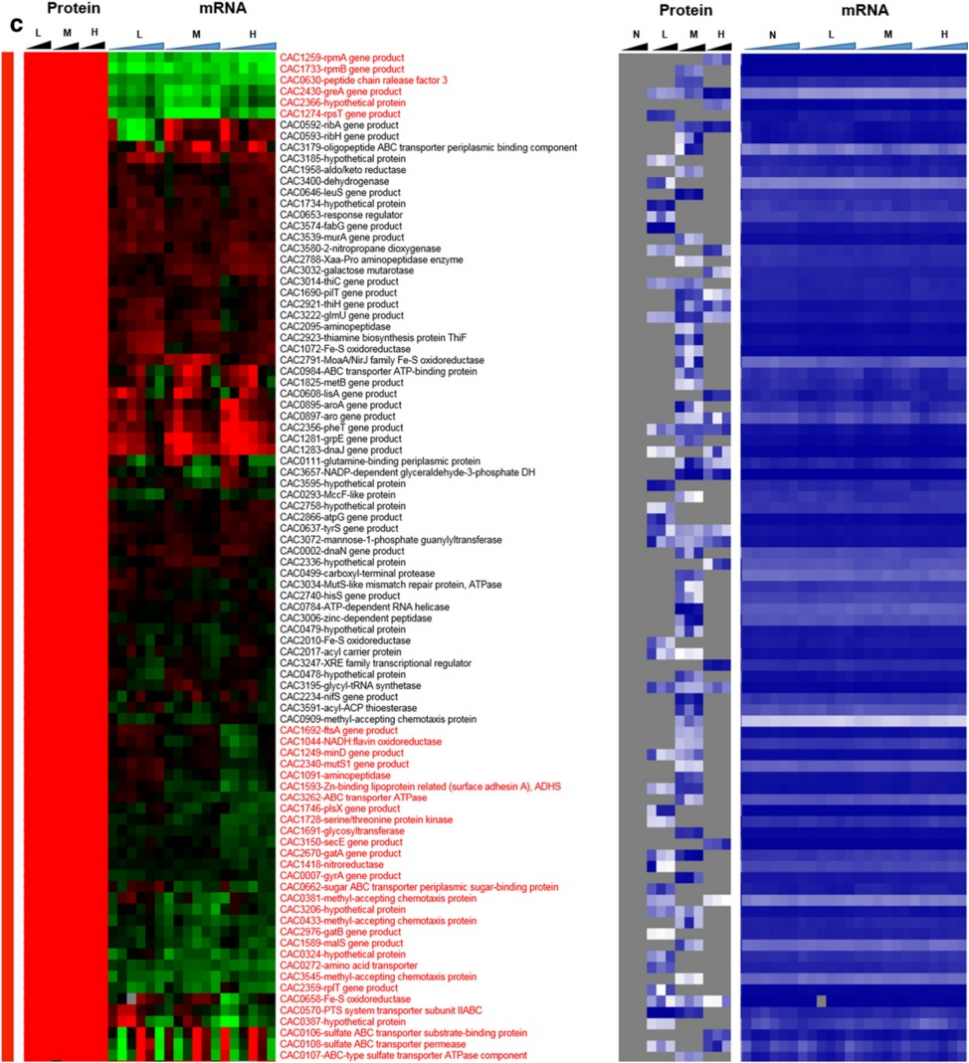
**(continued)** **c** proteins expressed only under stress (red vertical bar)

**Fig. 2:**
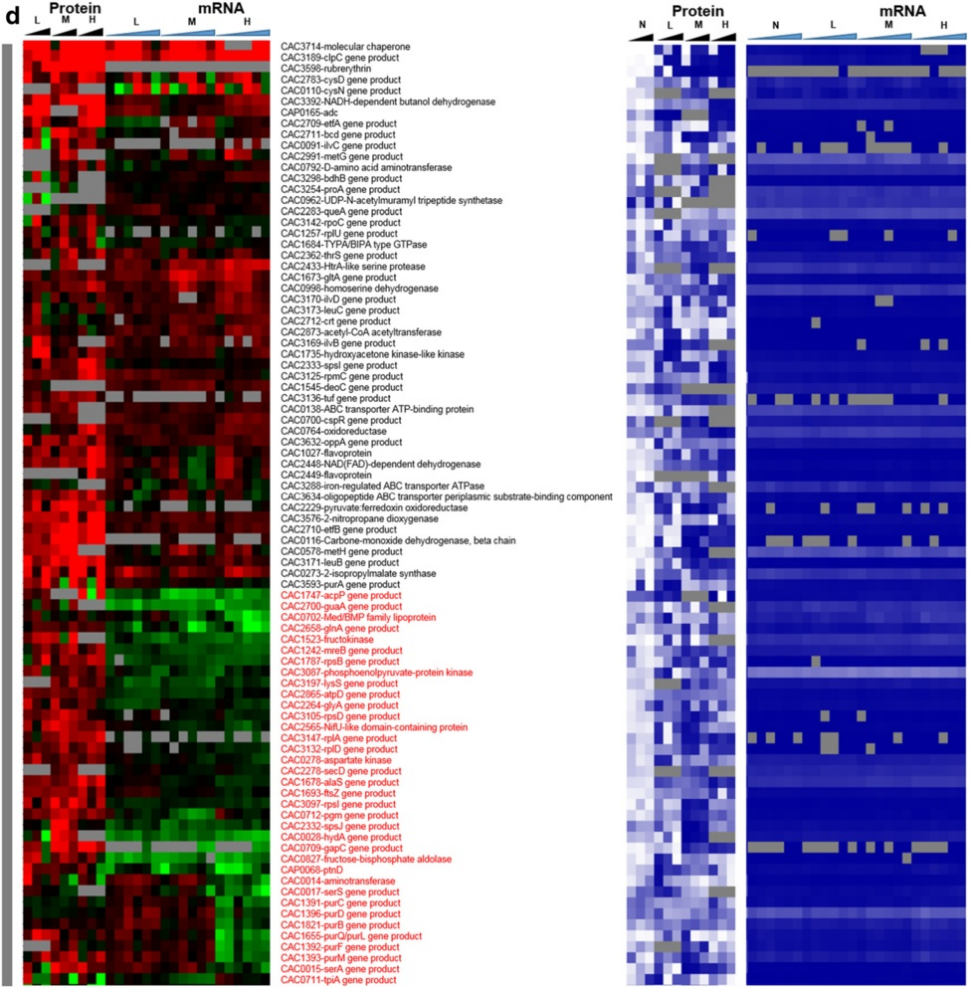
**(continued)** **d** significantly upregulated proteins under stress (grey vertical bar)

**Fig. 2:**
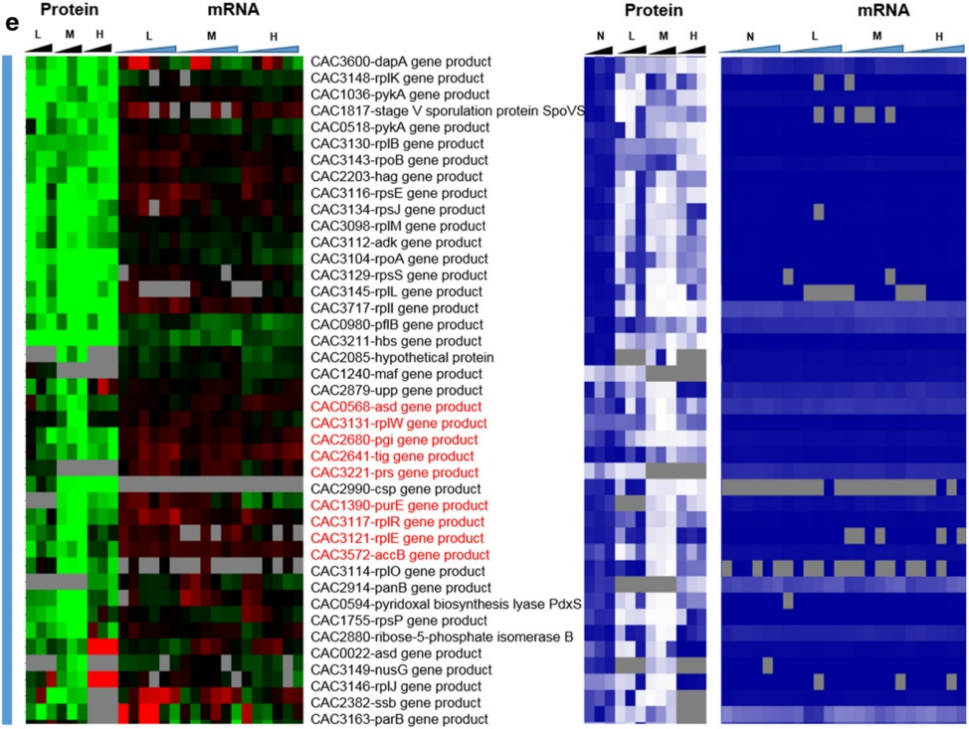
**(continued)** **e** significantly downregulated under stress (blue vertical bar). Genes that had a strong disagreement between mRNA and protein levels are represented in red font. Genes/proteins lacking expression (could not be detected with the methods used) and hence abundance ranking were represented by gray color

**Fig. 3:**
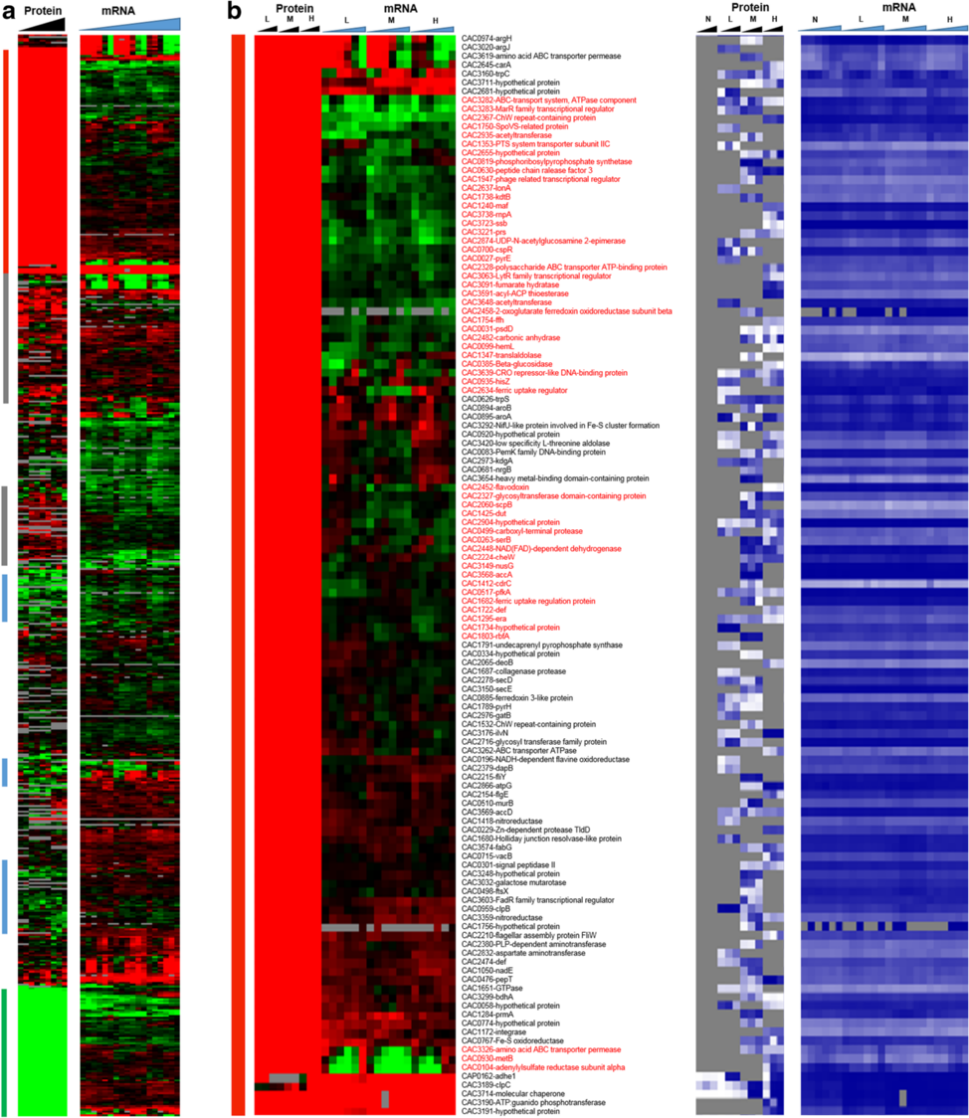
Heat map of proteomic and transcriptomic (microarrays) differential expression between butyrate stress and control condition. Differential gene expression of stress versus non-stress control is displayed in red-green and protein and mRNA abundance percentile ranking is shown in blue plots. **a** Butyrate stress proteome **b** proteins expressed under stress (red vertical bar). Expression scales are with panel (**e**)

**Fig. 3:**
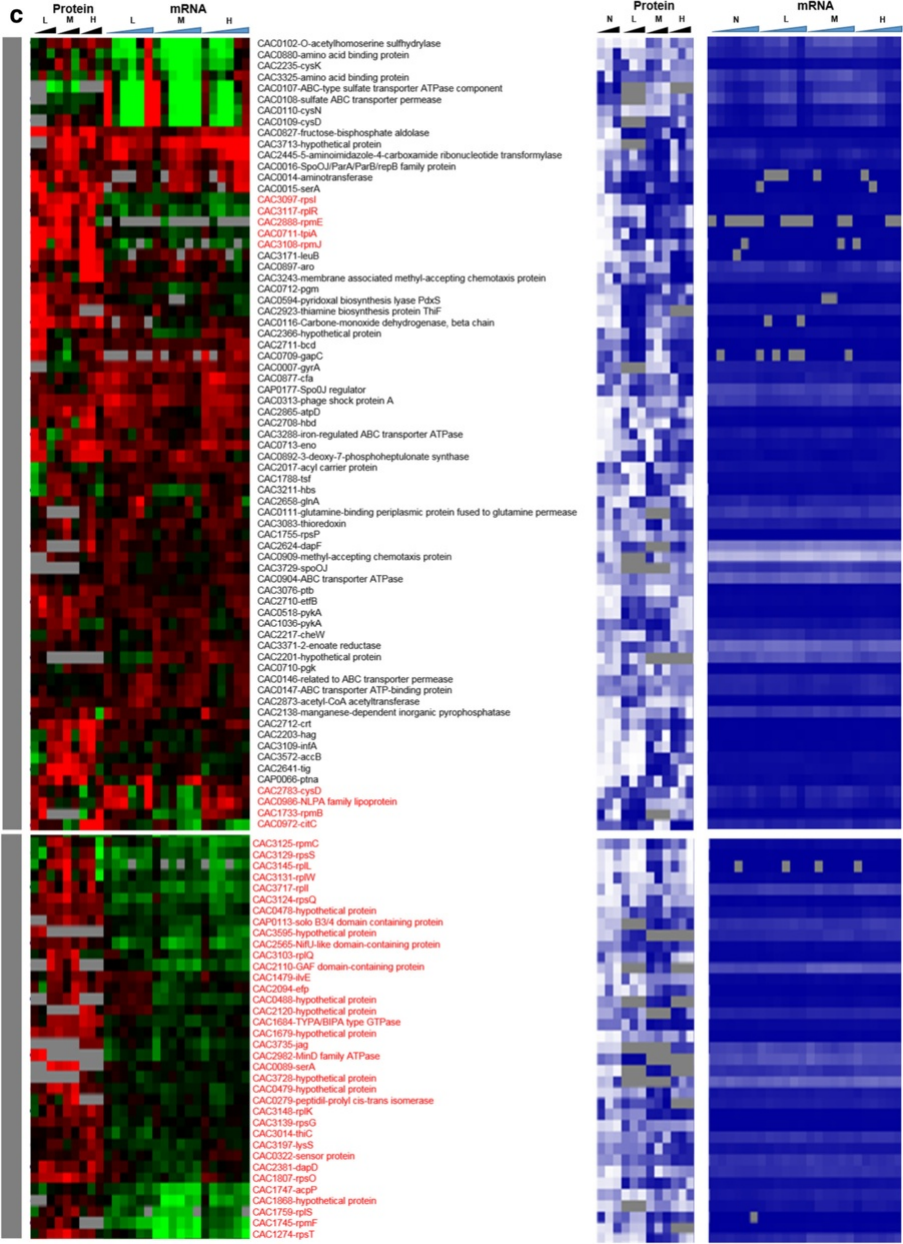
**(continued)** **c** significantly upregulated proteins under stress (grey vertical bar). Expression scales are with panel (**e**)

**Fig. 3:**
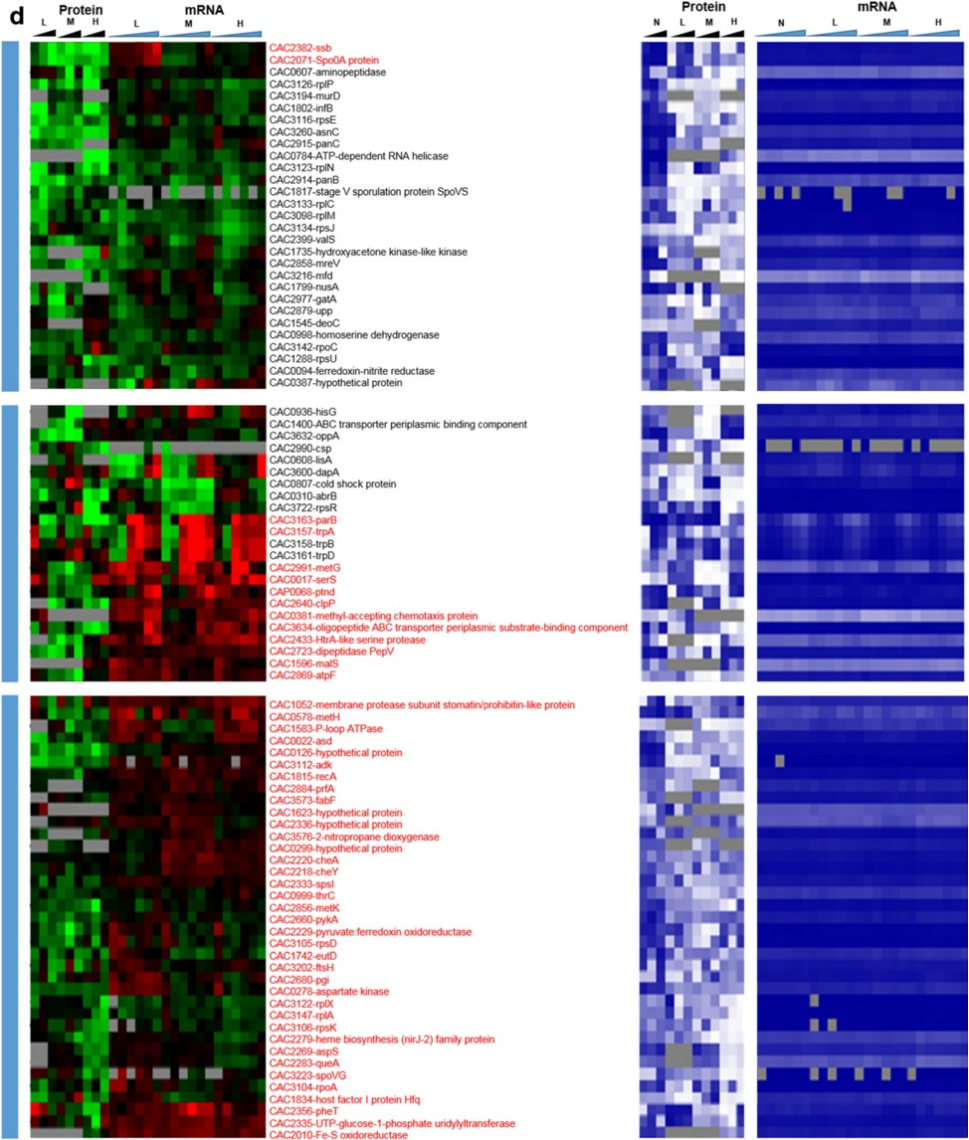
**(continued)** **d** significantly downregulated under stress (blue vertical bar). Expression scales are with panel (**e**)

**Fig. 3:**
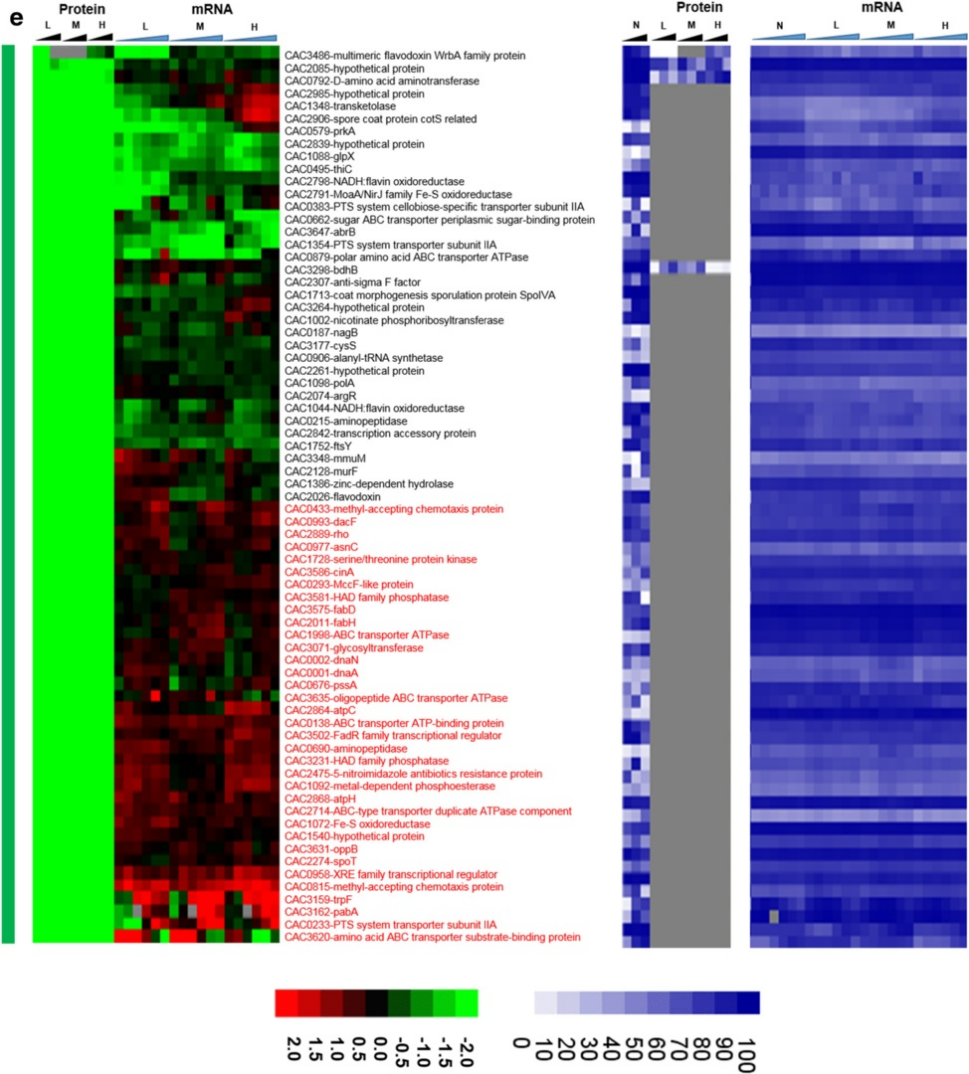
**(continued)** **e** proteins expressed only under non-stress control condition (green vertical bar). Genes that had a strong disagreement between mRNA and protein levels are represented in red font. Genes/proteins lacking expression (could not be detected with the methods used) and hence abundance ranking were represented by gray color

These data give a first glimpse of what will be reinforced below by analysis, namely that the butyrate stress proteome is considerably larger than the proteome of the butanol stress. This is based on the logical assumption that there is no bias from sample to sample in protein detection by the method employed here.

#### Differential expression of proteins under metabolite stress

Although, as already discussed, several studies have examined the metabolite stress response at the transcriptional level, relatively little is known about the stress proteome. As we shall show here, the detectable proteome displays distinct differences from the transcriptome, thus suggesting a more complex regulation that was anticipated from the transcriptomic studies. Analysis of differential protein expression was carried out by pairwise comparison of each time point between each stress condition and the corresponding non-stress condition using MeV’s SAM analysis (see Materials and methods); with the additional, typical requirement of a fold change ≥2.0, 306 proteins (Fig. 1) that were detected under both control and a given stress condition (low, medium or high) were considered for differential expression analysis under butanol stress. Among those, 243, 280, and 222 proteins were used for the analysis under low, medium, and high butanol stress, respectively, out of which 48, 76, and 75 proteins, respectively, were found to be significantly (fold change ≥2.0) differentially expressed. Similarly, under butyrate stress, 337, 344, and 357 proteins (a total of 402 proteins, Fig. 1) were used to analyze differential expression at low, medium, and high butyrate stress, out of which 55, 64, and 58 proteins, respectively, were found to be differentially expressed.

**Fig. 4:**
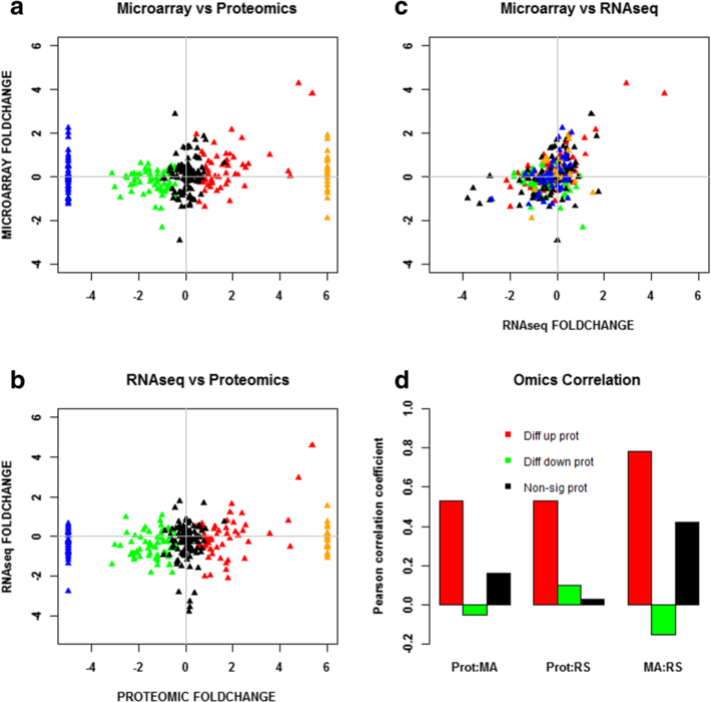
Comparison and correlation between proteomic and transcriptomic data under high butanol stress. **a** Microarray versus proteomic comparison. **b** RNAseq versus proteomic comparison. **c** Microarray versus RNAseq comparison. **d** Pearson correlation. All significant expressions are with respect to proteomic data only. Red: differentially upregulated proteins; green: differentially downregulated proteins; black: non-significant proteins; blue: proteins expressed only under non-stress control; orange: proteins expressed only under stress

**Fig. 5:**
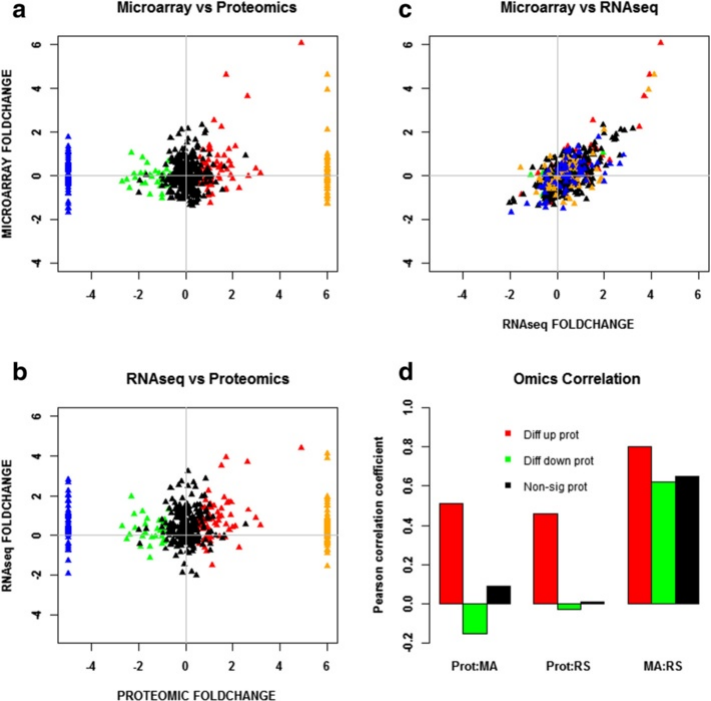
Comparison and correlation between proteomic and transcriptomic data under high butyrate stress. **a** Microarray versus proteomic comparison. **b** RNAseq versus proteomic comparison. **c** Microarray versus RNAseq comparison. **d** Pearson correlation. All significant expression are with respect to proteomic data only. Red: differentially upregulated proteins; green: differentially downregulated proteins; black: non-significant proteins; blue: proteins expressed only under non-stress control; orange: proteins expressed only under stress

Under both stress conditions, there was a set of proteins that were only under the control non-stress condition. These consisted of 44 and 67 proteins under butanol and butyrate stress, respectively (Table 1, Fig. 1, and Additional file 1: Figure S2). Similarly, several proteins were found to be expressed only under the stress condition and not under the control condition. These consisted of 90 and 120 proteins under butanol and butyrate conditions (Table 1, Fig. 1, and Additional file 1: Figure S2), respectively. These proteins/genes were classified as differentially expressed. Because it is not possible to calculate the fold difference for proteins expressed only under one (stress or control) condition, these two sets were assigned the maximum value observed for differentially (6.0) up- or downregulated (−5.0) proteins (Figs. 4 and 5). Some of the key proteins that were detected only under stress (which means that their expression levels under no stress were below detection limits) are summarized in Tables 2 and 3. These proteins belong to functional groups relevant to the stress response and the physiology of the cells, notably, to heat shock proteins (HSPs), UV stress response proteins, transcriptional regulators, response regulators involved in signal transduction, and chemotaxis proteins. Of note is that HSP proteins YacH and YacI in the *clpC* operon [16] were detected as expressed only under high butyrate stress but not under butanol stress.

**Table 1:** Differential expression analysis of proteomic data

A. Butanol stress						
	Low BuOH		Med BuOH		High BuOH	
Total proteins used for DE analysis	243		280		222	
	Up	Down	Up	Down	Up	Down
DE proteins (FDR 5 %)	23	40	20	63	53	63
DE proteins (FDR 5 %, fold change ≥2.0)	15	33	20	56	34	41
Proteins expressed only under stress	37		59		25	
Proteins expressed only under non-stress control	44					
B. Butyrate stress						
	Low BA		Med BA		High BA	
Total proteins used for DE analysis	337		344		357	
	Up	Down	Up	Down	Up	Down
DE proteins (FDR 5 %)	54	19	60	41	68	32
DE proteins (FDR 5 %, fold change ≥2.0)	38	17	38	26	36	22
Proteins expressed only under stress	43		70		61	
Proteins expressed only under non-stress control	67

**Table 2:** Key proteins expressed only under butanol stress condition but not under non-stress control condition

Proteins	Function
CAC1281 - GrpE	Heat shock response
CAC1283 - DnaJ	Heat shock response
CAC0381 - methyl-accepting chemotaxis protein	Chemotaxis
CAC0433 - methyl-accepting chemotaxis protein	Chemotaxis
CAC0909 - methyl-accepting chemotaxis protein	Chemotaxis
CAC3545 - methyl-accepting chemotaxis protein	Chemotaxis
CAC0653 - response regulator	Transcriptional regulator

**Table 3:** Key proteins expressed only under butyrate stress condition but not under non-stress control condition. A heat map for the expression of these proteins is shown in Additional file 1: Figure S8.

Proteins	Function
CAC3190 - YacI	Heat shock response
CAC3191 - YacH	Heat shock response
CAC0083 - UV resistance protein	Stress response
CAC1412 - PemK (MazF/MazE) family regulator	Transcriptional regulator
CAC2215 - FliY	Flagellar motor protein
CAC2224 - CheW	Chemotaxis

Under butanol stress, the functional groups containing proteins from the translational machinery (J), nucleic acid metabolism (F), and carbohydrate metabolism (G) were enriched in differentially downregulated proteins. Groups of energy metabolism (C) and amino acid metabolism (E) were enriched in differentially upregulated proteins (Fig. 1). Under butyrate stress, compared to butanol stress, more COG groups were enriched in upregulated proteins: COG groups C, E (amino acid metabolism and transport), F, G, O (post-translational modification, protein turnover, and chaperone functions), and T (signal transduction) were predominantly upregulated.

Downregulation of proteins of the translational machinery (COG category J) under butanol stress is consistent with the observed growth inhibition. In contrast, there was substantially less downregulation of proteins in COG category J under butyrate stress, whereby there was no growth inhibition observed. Other differences between butanol and butyrate stress were in the expression of proteins involved in energy production (COG category G) and nucleic acid metabolism (COG category F), which may explain the uninhibited growth of cells under butyrate stress, compared to growth inhibition under butanol stress. Additionally, the upregulation of these pathways under butyrate stress supports the reassimilation of butyrate as observed in transcriptomic studies [16, 12, 6, 7, 17, 18, 14].

### The global proteome versus the transcriptome under metabolite stress

#### Global comparison demonstrates more complexity that cannot be anticipated from the transcriptomic data

To probe whether proteomic and transcriptomic data agree in trends and patterns, we analyzed the data in several ways. First, we compared differential expression based on the proteomic data with the differential expression of the corresponding mRNAs using transcriptomic data from both RNAseq [15] and microarray analyses [16] (Fig. 2). The heat maps of this comparison make it possible to quickly identify genes/proteins, which are in agreement or not between mRNA and protein levels. The most interesting ones are the set of genes/proteins that are not in agreement between the two sets and are also important to physiology. In addition to differential expression, it is useful to also display a metric of a protein’s or mRNA’s expression level, which provides additional information for proteins/genes found expressed or differentially expressed under one condition but not in another, or at one level (protein or mRNA) but not the other. To this effect, as we have shown previously [15, 13] we employed the blue plots (heat maps), which display the relative abundance of a protein or mRNA with respect to total protein or mRNA level for given sample and time point. This relative abundance is displayed in percentile between 0 and 100 for the least and most abundant protein or mRNA, respectively. Twenty-three percent (102) and 31.5 % (186) of the proteins detected under butanol and butyrate stress, respectively, displayed clearly opposite patterns from the corresponding mRNA expression patterns. These conservative estimates represent a surprisingly large fraction of the expressed proteome and could not have been anticipated from previously published studies.

Among the proteins that were found to be expressed only under the control conditions and not under butanol stress (despite good mRNA-level expression as displayed in the blue heat maps), three proteins, coded by genes CAC0943-hisE, CAC0936-hisG, and CAC2065-deoB (Fig. 2b), are involved in histidine/purine metabolism. These three mRNAs were differentially upregulated, but no protein was detected under butanol stress. HisE and HisG encode for the protein product that catalyze the first two steps of histidine biosynthesis from PRPP (phosphoribosyl pyrophosphate), while deoB is involved in the generation of PRPP, a key metabolite precursor in histidine, purine, and pyrimidine metabolism. Other genes, which belong to this category, include two ribosomal proteins - CAC3115 (*rpmD*) and CAC1284 (*prmA*). Butanol stress inhibits cell growth, and it appears that a select set of ribosomal proteins are downregulated as a result of the severe inhibition of protein synthesis.

On the other hand, several proteins, which were transcriptionally downregulated, were found to be expressed under butanol stress but not under the control non-stress condition (despite good mRNA levels; blue heat map), i.e., they appear to be upregulated under butanol stress (Fig. 2c). Some of them, with known biological significance to stress response and/or cellular metabolism, include three ribosomal proteins (CAC1259-rpmA, CAC1733-rpmB, and CAC1274-rpsT), a transcriptional elongation factor, greA (CAC2430), a peptide chain release factor (CAC0630), and a hypothetical protein (CAC2366). These data and the data in the previous paragraph suggest that a different set of proteins is engaged for protein synthesis under butanol stress. Some of the other proteins in this group include ftsA (CAC1692), a cell division protein; mutS1 (CAC2340) recombination and DNA strand exchange inhibitor protein; and gyrA (CAC0007) DNA gyrase which are involved in cell division and DNA replication and/or transcription. These findings suggest that the cells are using posttranscriptional regulation to upregulate proteins necessary for repairing DNA damaged by the butanol insult, and continue cell division despite the severe, overall, growth inhibition. Butanol stress has a strong negative effect on membrane functionality as it affects the membrane fluidity [20], which in turn affects membrane transport and the transmembrane potential and **Δ**pH [21, 22]. Several ABC transporters and permeases (Fig. 2c) (CAC3262, CAC0662, CAC0272, CAC0570, CAC0108, CAC0107) for the transport of sugars, amino acids, and peptides were found to be translated under butanol stress but not under the control condition. These data suggest that the cells upregulate the expression of select membrane proteins, despite their transcriptional downregulation, aiming to deal with the chaotropic effect of butanol that inhibits membrane functions.

Figure 2d summarizes the proteins that were upregulated under stress. There was a small subset of proteins that were not in agreement with the transcriptional data, i.e., they were translationally upregulated from, overall, differentially downregulated mRNAs. These included several proteins from carbohydrate and energy metabolism, cell division (ftsZ), and ribosomal proteins (CAC1787, CAC3105, CAC3147, CAC3132). Again, these data suggest that the cells upregulate the translation of select sets of proteins aiming to ameliorate the inhibitory impact of butanol and despite the downregulation of these transcripts.

Focusing next to butyrate stress, several genes/proteins showed a disagreement between protein and mRNA expression patterns (Fig. 3). Among the proteins that were detected only under butyrate stress but not under control conditions (Fig. 3b), despite good mRNA levels (blue heat map), are proteins involved in several stress response pathways. These include proteins involved in DNA damage, repair, and replication (CAC3723 (*ssb*) - single-strand DNA binding protein); stress-related protease for protein quality control (IonA) and peptide chain release factor (CAC0630); cell division (Maf - septum formation protein; CAC1240); and ribonuclease P (CAC3738), which processes tRNAs and possibly sRNAs (Fig. 3b). Unlike butanol stress, proteins involved in histidine (HisZ) and PRPP metabolism (CAC0819, pyrE) were found to be upregulated (Fig. 3b), despite downregulated transcript levels. The histidine/purine metabolism involving PRPP displays opposite behavior in comparison to butanol stress. Furthermore, several ribosomal proteins and proteins of amino acid metabolism, such as lysine metabolism (Fig. 3c), were found to be upregulated at protein level despite lower transcript levels. Proteins involved in DNA replication, DnaA and DnaN, and fatty acid metabolism FabH and FabD (involved in initiation and elongation) were not detected under butyrate stress in comparison to the control condition (Fig. 3e), despite higher amounts of transcript (blue heat map), likely reflecting lower DNA and fatty acid biosynthesis rates under butyrate stress. In the same category (expressed under control but not under butyrate stress, despite good mRNA levels of expression; Fig. 3e) is CAC2889, the only annotated for the hexameric transcription termination factor Rho, with several additional roles in transcription and translation recently added to its repertoire, including the premature termination and degradation of spurious transcripts [23]. In *E. coli* and other well-studied model prokaryotes, Rho is apparently responsible for the termination of about half of the coded transcripts. This would suggest that, under butyrate stress, mRNAs dependent on Rho for termination are affected and probably improperly processed or unstable, thus resulting in massive and global changes in the RNome under butyrate stress as seems to be suggested by the data of Figs. 1, 2 and 3. Of note is that both the protein and the transcripts of two other transcriptional termination proteins (CAC3216-Mfd and CAC1799-NusA; Fig. 3d) that may offer alternate mRNA termination mechanisms [24] are also downregulated under butyrate stress.

Finally, we would like to draw attention to the signal recognition particle (SRP) GTPase (Ffh, coded by CAC1752), which, together with a small RNA non-coding RNA (the SRP-RNA), creates SRP. The SRP translocates the ribosomes to synthesize proteins in the membrane using the membrane-associated SRP receptor, SR, coded by *ftsY* (CAC1754). In our previous transcriptomic study [15], we found that the SRP-RNA was upregulated under butyrate stress. The transcriptomic data show low expression of *ffh* and *ftsY*, but the proteomic data revealed stress-specific expression of Ffh (Fig. 3b) under high butyrate stress. Surprisingly, the FtsY protein was not detected under butyrate stress although it was expressed (detected) at the control non-stress condition (Fig. 3e). These data may reflect the cell’s need to synthesize at higher rates a set of membrane proteins to ameliorate butyrate toxicity.

To sum this section, a first comparative analysis of the differential expression of proteins and their corresponding mRNA revealed complex regulation at post-transcriptional and/or translational levels of several pathways and programs of stress physiology. Those included programs for amino acid and nucleic metabolism; DNA replication, repair, and damage; the transcription and translation machinery, including transcriptional termination; as well the SRP-system proteins that could not have been detected using of only one type of omic data.

#### Probing protein- versus mRNA-level expression differences further

To further detail the extent to which the protein and mRNA data agree or disagree, and to bring the RNAseq-based mRNA data into the analysis, two-way comparisons of proteomic, microarray, and RNAseq data are presented in x-y scatter plots by grouping the proteins into five categories based on their pattern of expression: differentially upregulated, differentially downregulated, non-significant, expressed only under stress, and expressed only under control. For the last two groups, since expression of the proteins was detected only under one condition, as stated above, these two sets were assigned the maximum value observed for differentially (6.0) up- or downregulated (−5.0) proteins (Figs. 4 and 5) and are discussed separately. Pearson correlation coefficient among the proteomic, microarray, and RNAseq datasets were calculated for each of the three categories (differentially upregulated, differentially downregulated, and non-significant; due to the lack of standard deviation, Pearson correlation cannot be calculated for the last two categories for the proteins that were either expressed only under stress or only under control non-stress condition) and between the two transcriptomic datasets (microarray and RNAseq). These comparisons are summarized in Fig. 4 for high butanol and in Fig. 5 for high butyrate stress, respectively. High stress levels overall appear to accentuate the distinct features of each stress condition. The corresponding plots for low and medium stress levels are presented as Additional file 1: (Figures S3–S6). Overall, the correlation between the two transcriptomic datasets is far superior to the proteomic-transcriptomic comparisons. The microarray versus RNAseq transcriptomic comparison shows extremely high correlation among the differentially upregulated genes/proteins (Figs. 4 and 5) but a lower correlation for the genes/proteins that were differentially downregulated or non-significantly expressed for butanol stress (Fig. 4). These disagreements arise due to the differences in technology between microarray and RNAseq along with transcript abundance and amplification of transcripts/cDNA during RNAseq library preparation [25].

A high correlation between mRNA and protein levels was observed among the differentially upregulated proteins and a low correlation between mRNA and protein level for the differentially downregulated and non-significantly regulated proteins. As discussed above, the majority of proteins belonging to the differentially upregulated group belonged to proteins of the post-translational modification, protein turnover, and chaperone systems. Differentially downregulated proteins have the lowest correlation between proteomic and transcriptomic data. This lack of correlation can be attributed to two key factors: inefficiency of the translational machinery under stress and post-transcriptional regulation of the transcripts, such as by regulatory non-coding small RNAs (sRNAs), which have been identified to be involved in this stress response [15]. We note that issues of mRNA and protein stability and degradation cannot be responsible for the observed differences as these two processes are already taken into account in the temporal “snapshots” of these omic data (Figs. 2 and 3). To further probe the basis for these differences, we examined the data from the regulon and program point of view, as well as from the point of view of the structural features of the corresponding mRNAs. The former would argue for an evolutionary basis for the observed disagreements between protein and mRNA levels, while the latter could explain how these differences can be explained by molecular regulation based on mRNA features. First, we analyzed key regulons and programs that likely play an important role under stress in this organism [12, 6, 17, 18, 14, 16].

### The stress proteome versus transcriptome of key regulons and the translation program

#### HrcA and CtsR regulons

The HrcA and CtsR regulons are two core stress-responsive regulons involved in the canonical stress or heat shock (HSP) response. These two regulons were precisely identified and detailed based on transcriptomic data and bioinformatics analyses [16]. The HrcA regulon, which consists of eight genes in four operons, was transcriptionally (based on both microarray and RNAseq data) strongly upregulated (Fig. 6a, b) in response to both stresses. The proteomic data show that, under stress, and especially butanol stress, these upregulated transcripts were overall poorly translated or that these proteins were unstable. Under butyrate stress, proteins for six of the eight HrcA genes were detected, three of which showed good correlation between mRNA and protein level. Under butanol stress, five of the eight proteins were detected and two to three showed reasonable correlation with mRNA patterns. GrpE and DnaJ were detected as expressed only under butanol stress but not under control conditions (Fig. 6a).

**Fig. 6:**
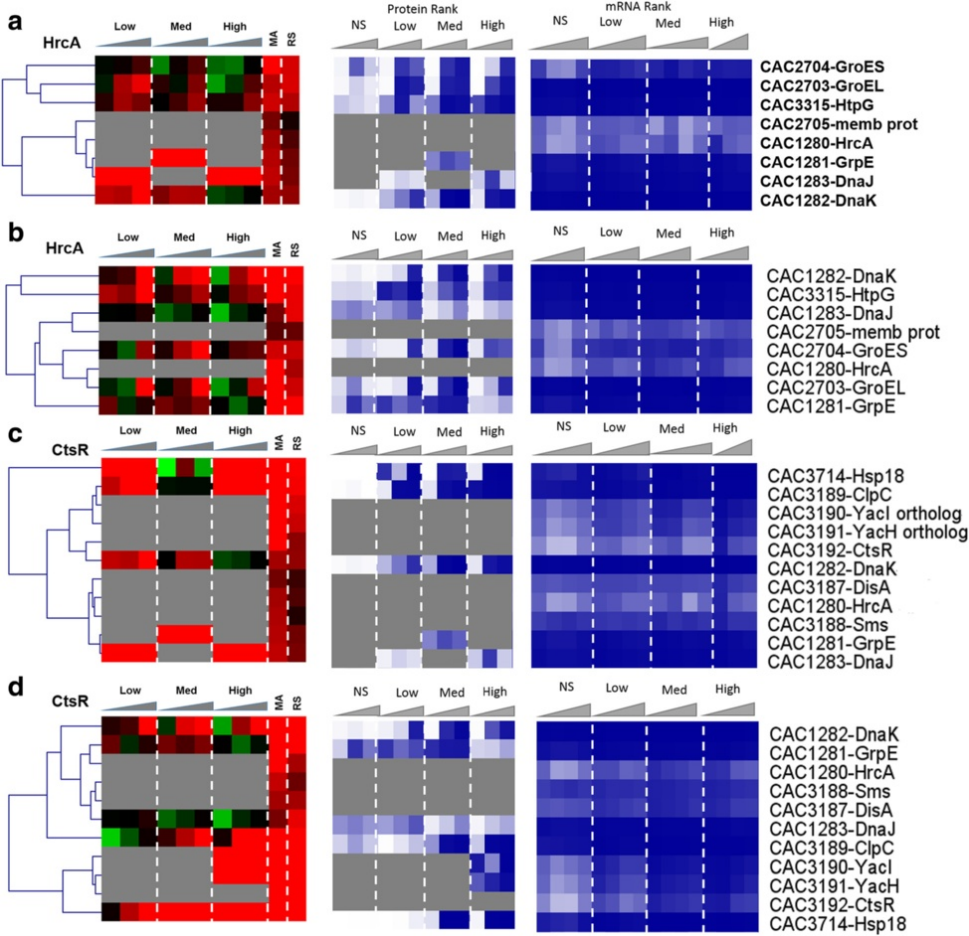
Regulon analysis. Comparison of the proteomic and transcriptomic expression of HrcA regulon: **a** butanol stress, **b** butyrate stress; CtsR regulon: **c** butanol stress, **d** butyrate stress, and Rex regulon. Expression scales are with panel (**e**)

The CtsR regulon consists of 11 genes (Fig. 6c, d), which were highly upregulated at the mRNA level, under both butanol and butyrate stress. Seven and five of the corresponding proteins were detected under butyrate and butanol stress, respectively. Fewer among those showed any reasonable agreement with mRNA patterns. While the proteins of the two transcriptional regulators (HrcA and CtsR) were not sufficiently abundant to be detected by the proteome method used but based on the current and prior transcriptomic studies [12, 6, 7, 13–16], one would have expected the proteins (heat shock and general stress proteins) regulated by these two transcriptional regulators to be detected under stress due to their stress-induced expression. Some of the observed patterns are also surprising for additional reasons. For example, YacI and YacH, two genes in the ClpC operon, were detected as expressed only under high butyrate stress and not at all under butanol stress. In contrast, ClpC was detected as expressed under most stress conditions. We note that the two regulons share a few common members, notably HrcA, GrpE, DnaK, and DnaJ, which are located in the same genomic locus and organized in operons that may share common regulatory features [16]. Still, the protein patterns detected here are very different among these proteins and especially so between the two stress conditions. These data suggest strong translational regulation or protein instability for the CtsR and HrcA regulon proteins.

#### The Rex regulon is important in the response to butyrate but not butanol stress

Rex (CAC2713) is the redox sensor transcription (repressor) factor. Its regulon in *C. acetobutylicum* was identified using phylogenetic foot printing analysis [16]. The Rex regulon plays an important role in regulating the overall redox balance, NADH/NAD^+^ levels, ATP synthesis, and electron transport, thus regulating the central carbon and energy metabolism, and is especially important in solventogenic clostridia as it has been shown to regulate the shift from acidogenesis to solventogenesis [26].

Both the *rex* transcript and Rex protein were found to be downregulated under butyrate stress (Fig. 6e), thus displaying good correlation between mRNA and protein levels. We were happily surprised to be able to detect Rex at the protein level, given that it is a regulator typically expressed at lower levels. Under butyrate stress, although only 20 of the 33 proteins of the Rex regulon were identified by our proteome method (Fig. 6e), there was overall in good correlation between protein and transcript levels (based on both the RNAseq and microarray data). Notably, the proteins/genes involved in ATP synthesis (CAC2864–CAC2871), electron transport (EtfA, EtfB), butyrate production (Thl, Crt, Bcd), butyrate assimilation to form butanol (AdhE1/Aad), amino acid metabolism (aminotransferase and SerA), and carbon and energy metabolism (GapC, aldolase) displayed an overall good correlation between mRNA and protein levels. It is interesting to note that although all three genes (namely the genes the *sol* operon (*adhE1*/*aad*-*ctfA*-*ctfB*)) involved in solvent production were upregulated at the mRNA level, the protein of only the first gene, *aad*, was detectable under butyrate stress and found to be also upregulated. This would suggest preferential translation of selected genes in an operon under stress apparently due to post-transcriptional regulation. As shown from the physiological metabolite data (Additional file 1: Figure S1), the cultures stressed with butyrate produced more butanol with corresponding increase in butyrate stress but on the other hand, acetone production decreased with increasing in butyrate stress.

**Fig. 6:**
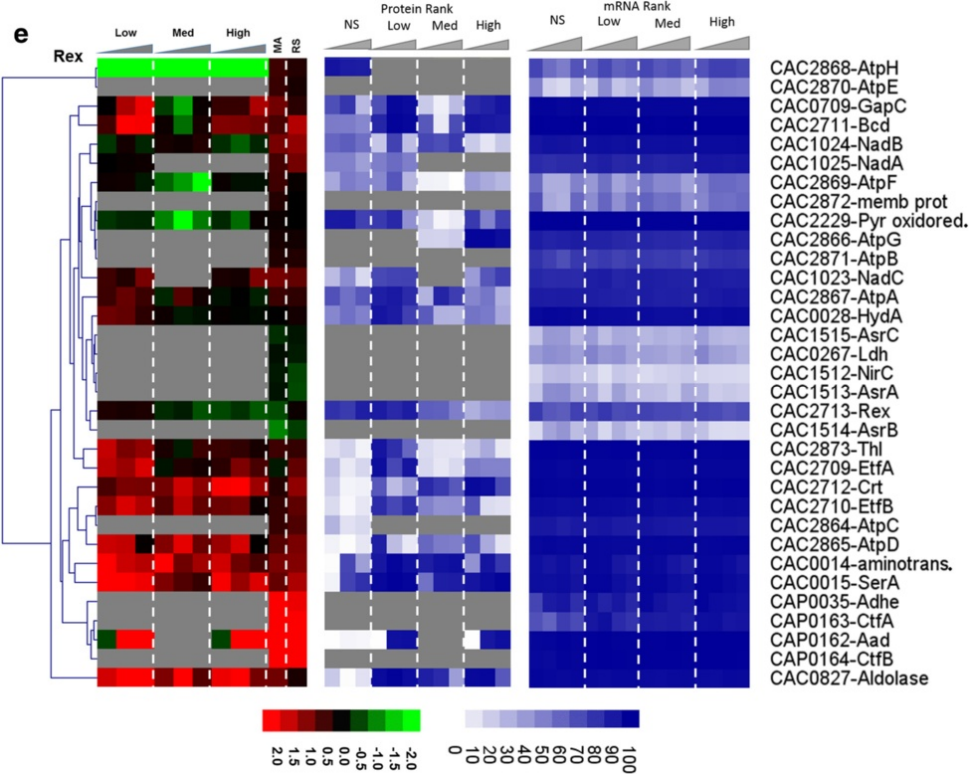
**(continued)** **e** butyrate stress. The microarray (MA) and RNAseq (RS) values are displayed as average fold change across all time points. Proteins/genes are listed by genomic number and an abbreviated accepted name. Genes/proteins lacking expression were represented by gray color. Differential gene expression of stress versus non-stress control is displayed in red-green and protein and mRNA abundance percentile ranking is shown in blue plots

Unlike the butyrate stress, the *rex* gene was not affected by butanol stress, and its protein did not display a clear pattern of expression. It is not surprising then that fewer proteins (14) of the Rex regulon were identified at the protein level under butanol stress, and that there was a poor correlation between protein and mRNA levels. The only interesting observation is that although all three transcripts of the *sol* operon were upregulated under butanol stress, none of the proteins was detectable by our method, again highlighting the importance of post-transcriptional regulation. We conclude that the Rex regulon has no important role in butanol stress.

#### Ribosomal and related proteins of the translation machinery proteins and the differential expression of leaderless transcripts

To assess the impact of metabolite stress on the translation machinery, the protein and transcripts levels of all 74 annotated ribosomal proteins and seven accessory proteins were analyzed (Fig. 7). The accessory proteins include GTPases that are essential for the translational machinery. Twenty of the ribosomal proteins and four of the accessory proteins remained undetected in the proteomic analysis under either butanol or butyrate stress. Among the detected 57 proteins, the majority was downregulated under butanol stress despite higher transcriptional levels. Overall, there was poor correlation between protein and mRNA levels for these proteins, with only a few exceptions. Under butyrate stress, which exhibited little or no inhibition of growth with respect to the non-stress control condition, a large fraction of the ribosomal proteins were upregulated and only a few downregulated. In contrast, under butanol stress, which leads to growth inhibition (Additional file 1: Figure S1), the majority of the ribosomal proteins were found to be downregulated, and this makes logical sense. It is interesting to note that a few ribosomal proteins were upregulated, including those coded by CAC1787, CAC3097, CAC3125, CAC3132, and CAC3147 thus suggesting that the cells may employ an alternate set of protein for protein synthesis under stress. Along the same lines, under butyrate stress, there were a few translation-related protein that were not only upregulated under some level of butyrate stress but those were proteins not detected under non-stress conditions and include CAC1803, CAC1284, and CAC1295. A specific mechanism that results in the use of an alternate translational machinery under stress in *E. coli* [27, 28] engages the toxin-antitoxin (MazF-MazE) system that generates leaderless transcripts and a subpopulation of alternate ribosomes to translate the leaderless transcripts. In leaderless transcripts, the transcriptional start site and the translational start sites are the same, and thus they lack a ribosomal binding site, RBS. No other stress-specific mechanisms regarding changed ribosomal composition have been reported in the literature. This prompted us to examine the existence and possible differential transcription of leaderless transcripts.

**Fig. 7:**
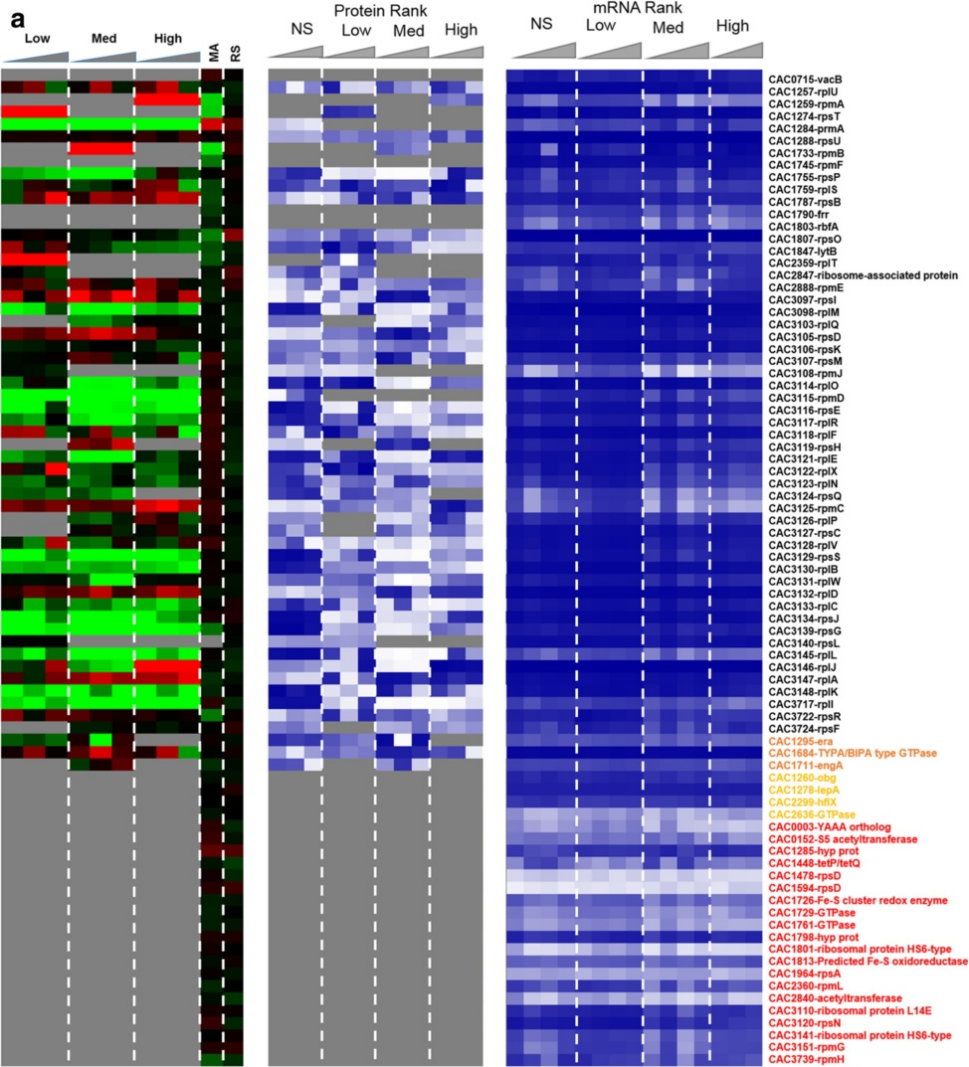
Effect of stress on translational machinery. Comparison of the proteomic and transcriptomic expression of ribosomal proteins **a** butyrate stress. Expression scales are with panel (**b**)

**Fig. 7:**
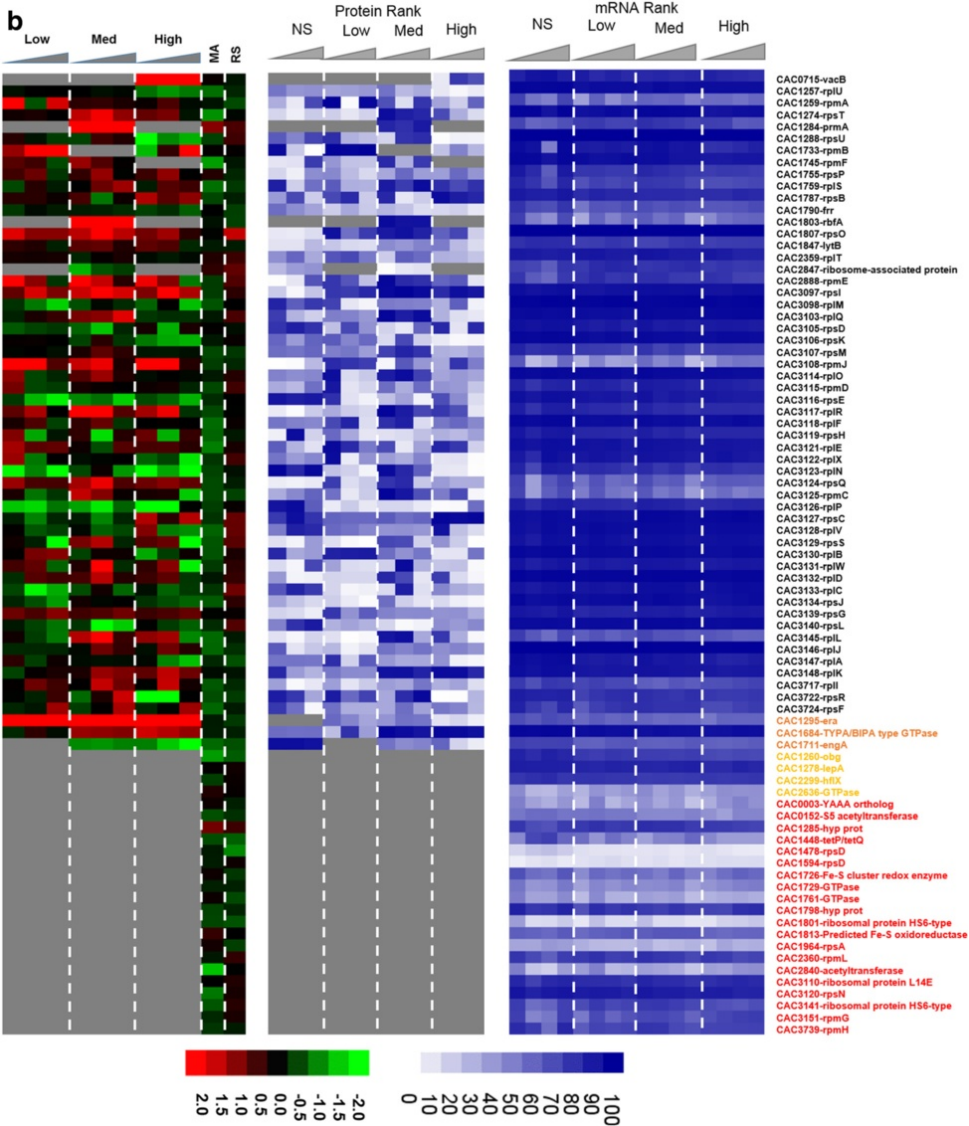
**(continued)** **b** butanol stress. The microarray (MA) and RNAseq (RS) values are displayed as average fold change across all time points. Proteins/genes are listed by genomic number and an abbreviated accepted name. Genes/proteins lacking expression were represented by gray color. Differential gene expression of stress versus non-stress control is displayed in red-green and protein and mRNA abundance percentile ranking is shown in blue plots. Legend font color: orange: accessory GTPases detected in proteomics; yellow: accessory GTPases NOT detected in proteomics; red: ribosomal proteins NOT detected in proteomics

Leaderless transcripts are either naturally coded on the chromosome as leaderless or are generated by some specific mechanism that trims canonical mRNAs that contain a RBS. Using the strand-specific RNAseq data, we identified 212 leaderless transcripts among the genes corresponding to the proteins identified by proteomic analysis. These leaderless transcripts are, by definition, either monocistronic operons or the first gene of a polycistronic operon, based on the transcriptional operon organization of *C. acetobutylicum* [29]. Further, we identified 102 transcripts under butanol stress and 120 transcripts under butyrate stress, as being leaderless (Additional file 1: Figure S7). Among these leaderless transcripts, 22 and 37 corresponded to proteins that had a strong disagreement between proteins and mRNA levels (Figs. 2 and 3) under butanol and butyrate stress, respectively. Nine transcripts which had 5′UTR under non-stress condition were found to be leaderless under both butanol and butyrate stress. On the other hand, 28 and 29 leaderless transcripts under non-stress condition were found to contain 5′UTR with a RBS under butanol and butyrate stress. These results confirm the presence of leaderless transcripts in this organism along with differential transcriptional start sites under stress. Leaderless transcripts have been identified in several bacterial transcriptomes [30–33]. A general mechanism, identified in *E. coli* (but not in *C. acetobutylicum* yet) for the translation of leaderless transcripts, involves the initiation factors 2 (IF-2) and 3 (IF-3) [34], where a higher IF-2/IF-3 ratio would favor the transcription of leaderless transcripts, such as has been observed under a cold shock in *E. coli* [34]. The *C. acetobutylicum* IF-3 protein (CAC2361) is expressed well under no stress conditions but was undetectable under butanol stress (Fig. 2b), thus presumably leading to a higher IF-2/IF-3 ratio. IF-2 (CAC1802) was downregulated under butyrate stress (Fig. 3d). In this light, we re-assessed possible roles of the downregulated ribosomal proteins (Fig. 7) in transcriptional elongation and termination. In *E. coli*, one well-known protein among those, S10 (coded by the CAC3134 (*rpsJ*) gene in *C. acetobutylicum*) is part of a transcriptional antitermination system with the transcriptional elongation factors NusA and NusG [24] (these are coded by CAC1799 and CAC3149, respectively, in *C. acetobutylicum*). This system interacts with the Rho protein to control intragenic transcriptional Rho-dependent termination [23]. The systematic downregulation of the S10 protein under both butyrate and butanol stress suggests changes in transcriptional termination under stress, and this is further supported by the finding that the expression of the Rho protein (which plays also a role in the coupling between translation and transcription [23]) is strongly downregulated under butyrate stress as already discussed.

As discussed above, in addition to naturally coded leaderless transcripts, in *E. coli*, the MazE/MazF system has been found to generate leaderless transcripts and facilitate their translation by generating modified ribosomes [27, 28]. An ortholog of the MazF-MazE system has been annotated in *C. acetobutylicum* (CAC0493–CAC0494). These two genes do not display any changes at the transcriptional level and were not detected by our proteomic analysis, thus reflecting a low level of protein expression. Nevertheless, their role in clostridial stress response deserves further investigation. It is not unlikely, however, that other mechanisms exist to trim canonical mRNAs into leaderless transcripts.

### What drives the disagreement between mRNA and protein levels for such a large fraction of the genome?

#### Mechanisms involving 5′UTR length and RBS strength

A number of studies across different species and organisms have shown lack of good correlation between mRNA and protein levels [35–37]. Such lack of correlation could be explained by different mechanistic hypotheses all based on structural features of mRNAs that would enable complex physiological regulation, which here is stress-responsive regulation. mRNA translation efficiency can be affected by the strength of the ribosome binding site (RBS) [38], the use of rare codons in the mRNA [39, 31], or regulation of the translation-initiation process [31], such as accessibility of the mRNA by the translation proteins as might be affected by special features of the 5′ untranslated region (5′UTR) of the mRNA [40]. Special features of the 5′UTR could include self-bending of the 5′UTR preventing accessibility to the RBS, thus requiring specific sRNAs to prevent bending/looping and thus allow translation [41]. Other regulation could include binding of specific sRNAs near the RBS thus preventing translation [42]. Transcripts engaging such regulation are typically characterized by longer 5′UTRs.

Based on these mechanistic possibilities, we probed three hypotheses as discussed below. This required that we compute the length of the 5′UTR of all transcripts and also of the RBS strength. Transcriptional start sites (TSSs) along with 5′UTRs were determined using our strand-specific RNAseq data (see Materials and methods for more details). A total of 389 5′UTRs were determined for transcripts encoded in the chromosome, and 24 5′UTRs were determined for the transcripts in the megaplasmid pSOL1. In *C. acetobutylicum*, the median length of the 5′UTR is 42 for the first genes of an operon, which is similar to the numbers reported in other bacterial species [30, 43]. Thus, in view of the existence of many leaderless transcripts, 5′UTRs longer than 42 would likely contain regulatory elements that could affect mRNA stability and translation. We calculated RBS strength using Prodigal [44], a software that determines translational start sites and assigns a score to the RBS based on the motif and the spacer, which is the distance between the RBS motif and the start codon. These scores varied between −18.92 for extremely weak or no RBS and 12.87 for strongest RBS. The average score for the RBS was 7.09. One hundred ninety-three genes were found by the Prodigal algorithm to contain no RBS. Thus, RBS strength values above 7 or 8 could be viewed as indicating strong translational possibilities. Next, we examined three mechanistic hypotheses.

The first hypothesis is that there is a set of mRNAs, which is translated inefficiently due to regulation by stress-responsive mechanisms affecting the accessibility of the mRNA for protein translation, such as the need for sRNAs to enable translation [41, 15]. These mRNAs then should display an anti-correlation with high mRNAs but relatively low protein levels under all conditions alike, stressed or non-stressed control conditions. An analysis of the data based on this hypothesis led to a null set, indicating that mRNAs with such anti-correlation were not observed under all conditions in this study.

The second hypothesis is that there is a set of mRNAs, which are translated inefficiently as a result of regulation from sRNAs or other related mechanisms under stress but not under non-stress condition. These mRNAs should have an anti-correlation with high mRNA levels (fold change >2.0) and low protein levels. We limited the search to medium and high level of stress conditions. No such proteins were found under either butanol or butyrate stress. We modified this hypothesis as discussed in the next section.

Our third hypothesis is that there is a set of mRNAs that display extraordinarily high translation efficiency and/or protein stability under stress conditions with low mRNA levels but high protein levels. We discovered 11 and 12 proteins/genes (hypothesis 3) under butyrate and butanol stress, respectively (Tables 4 and 5). These proteins were found to be differentially expressed, but their corresponding transcripts were not differentially expressed; rather, they displayed non-significant differential expression. Among the 11 proteins identified under butyrate stress, four (CAC1393 - PurM from purine metabolism, CAC3713 - hypothetical protein, CAC3097 - RpsI ribosomal protein, CAC2641 - trigger factor) and five (CAC0897 - aro, CAC3171 - LeuB, CAC3243 - chemotaxis protein, CAC0827 - fructose bisphosphate aldolase, CAC0972 - isocitrate dehydrogenase) proteins were found at medium and high stress, respectively, while two (CAC3598 - rubrerythrin and CAC0316 - ArgF/I) were found under both medium and high stress. Similarly, among the 12 proteins identified under butanol stress, three (CAC2229 - pyruvate:ferredoxin oxidoreductase, CAC0578 - MetH, CAC3392 - Bdh) and five (CAC2709 - EtfA, CAC0022 - aspartate semialdehyde dehydrogenase, CAP0165 - Adc, CAC2333 - SpsI, CAC3146 - RplJ) were identified under medium and high stress, respectively, while four (CAC3171, CAC3598, CAC0116 - CODH β subunit, and CAC2710 - EtfB) were found under both medium and high butanol stress. Examination of the 5′UTR from transcriptomic data and RBS strength scores (Tables 4 and 5) shows that virtually all genes have high (much above the average) RBS scores and about half of them has also long (much above the average) 5′UTRs, which could account for the high protein levels despite low mRNA levels. Notable among these proteins is rubrerythrin as discussed next.

**Table 4:** Key genes with high protein levels from low mRNA levels—butyrate stress

Gene	Function	5′UTR	RBS score
*CAC3713*	Hypothetical protein	96	12.87
*CAC2641*	Trigger factor	33	12.87
*CAC0897*	Aro	13	9.25
*CAC3171*	LeuB	30	7.68
*CAC3243*	Chemotaxis protein	15	12.38
*CAC3598*	Rubrerythrin	96	12.87

Genes are in italics to indicate their differential proteomic upregulation

**Table 5:** Key genes with high protein levels from non-significant mRNA levels—butanol stress

Gene	Function	5′UTR	RBS score
*CAC0116*	CODH β subunit	84	12.87
*CAC2229*	PFOR	76	9.25
*CAC2333*	SpsI - dTDP-glucose pyrophosphorylase	104	12.87
*CAP0165*	Adc - acetoacetate decarboxylase	63	12.09
*CAC3171*	LeuB	30	12.38
*CAC3598*	Rubrerythrin	96	12.87

Genes are in italics to indicate their differential proteomic upregulation

Rubrerythrin has been reported to act as an oxidative stress response protein in *C. acetobutylicum* [45, 46] and other *Clostridium* species [47, 48]. Rubrerythrin is viewed as a scavenger of dioxygen by acting as electron transport intermediary [48, 49]. In *C. acetobutylicum*, there are two copies of the gene (CAC3597 and CAC3598) and three other proteins, with an identity of 50 %, namely, CAC2575, CAC2778, and CAC3018, which are also annotated as rubrerythrins. Expression from our proteomic data mapped with the two copies of CAC3597–CAC3598, forming an operon in an arrangement viewed as a gene duplication [46]. Our proteomic data show that it is upregulated an average of 16-fold under butyrate stress, and up to 40-folds under butanol stress (Figs. 2, 3, 4 and 5). This clearly suggests the possibility of post-transcriptional regulation and/or high protein stability. Furthermore, using our strand-specific RNAseq data, CAC3598, the first of the two rubrerythrin genes in the operon, was found to contain a long 5′UTR of 96 nucleotides (Table 3). RNA secondary structure (Additional file 1: Figure S9) of the 5′UTR on its own and 5′UTR with varied length of the ORF (40 bases, 84 bases, and the full ORF) displayed the presence of loop of the 5′UTR and the protein coding region. The access to RBS progressively decreased and became more stable (denoted by an increase in the free energy) with an increase in the length of the ORF and hence preventing the binding of ribosomes for translation [50]. This inhibition is usually removed by the expression of a specific non-coding sRNA, as has been reported in other organism [51] and requires further investigation in *C. acetobutylicum* with respect to their stress-specific sRNome [15].

#### Revising and revisiting hypotheses 2 and 3

**Fig. 8:**
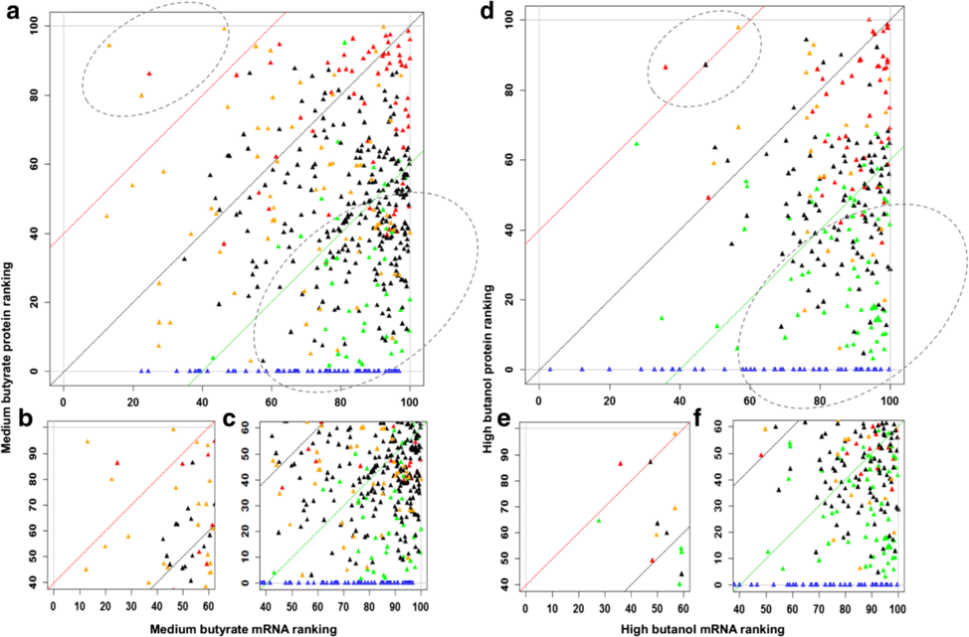
Comparison of the transcript-protein abundance percentile ranking. (**a**, **b**, **c**) medium butyrate stress and (**d**, **e**, **f**) high butanol stress. X-axis represents the average percentile transcript (RNAseq) abundance ranking under stress, and y-axis represents average percentile transcript abundance ranking under non-stress control. Red: differentially upregulated proteins; green: differentially downregulated proteins; black: non-significant proteins; blue: proteins expressed only under non-stress control; orange: proteins expressed only under stress

We revisited hypotheses 2 and 3, by comparing the abundance of mRNA and protein. As hypotheses 2 and 3 compared differential expression of mRNAs and their corresponding proteins, the revised hypotheses compared the actual abundance of the mRNAs and proteins among the entire transcriptome and proteome at a given stress condition. These modified hypotheses are if low abundance mRNAs express highly abundant proteins or if highly abundant mRNAs have low expression of their encoded proteins. To examine these revised hypotheses, the average transcript abundance percentile ranking for all the identified proteome under each stress condition was plotted against the average protein abundance percentile ranking (Fig. 8 and Additional file 1: Figures S10–S13). Protein expressed from transcripts with a percentile ranking as low as 2 was observed under butanol stress while the minimum average transcript abundance percentile ranking of the observed proteins under butyrate stress was 10. If a linear relationship between the mRNA and protein level were to exist, the abundance ranking plot would contain the differentially upregulated proteins at the top right corner with highly abundant transcripts and the differentially downregulated proteins on the bottom left with their low abundance transcripts. Nevertheless, this is not true, as the relationship between mRNA and protein is not linear. Hatzimanikatis and Lee have reported that the non-linear relationship between mRNA and protein level is driven by several factors such as mRNA stability and degradation, translation efficiency for a given protein along with the presence of post-transcriptional regulation [52]. Hence, we focused on those proteins that displayed such non-linear relationships (Fig. 8) and analyzed them further. These analyses were limited to medium butyrate stress and high butanol stress as these conditions showed more such interesting discordances.

Under medium butyrate stress, four proteins (Table 6) had low mRNA abundance ranking (<60) but had higher protein percentile ranking (>80). CAC3211 was differentially upregulated, while the other three proteins were found to be expressed only under butyrate stress. As these proteins were expressed from relatively less abundant mRNAs, their corresponding ribosome binding sites (RBSs) were analyzed. All the four genes, CAC3211, CAC3654, CAC0819, and CAC1425, contained high RBS scores near the upper limit of 12.87, namely 12.87, 12.38, 12.38, and 11.11, respectively. The presence of such strong RBSs in these proteins can explain the high protein levels despite low mRNA levels. Similarly under high butanol stress (Fig. 8, Table 7), CAC0108 (sulfate ABD transporter permease), CAC0091 - IlvC, and CAC1301 - hypothetical protein had higher protein expression and lower mRNA levels as well as very high RBS scores at or near the upper limit of 12.87.

**Table 6:** Genes with less abundant (<60 percentile rank) mRNA and highly abundant (>80 percentile rank) proteins—butyrate stress

Gene	Function	RBS score
**CAC1425** ^a^	Dut - dUTP hydrolase	11.11
**CAC3654** ^a^	Heavy metal binding	12.38
**CAC0819** ^a^	PRPP syn.	12.38
*CAC3211* ^b^	DNA binding protein	12.87

Genes are in italics to indicate their differential proteomic upregulation, while bold represents proteins that were expressed only under butyrate stress and lacked expression under control condition

^a^5′UTR was not determined due to lack of sufficient data (reads)

^b^CAC3211 had a 5′UTR of 19 under butyrate stress but was leaderless under non-stress condition

**Table 7:** Genes with less abundant mRNA (<60 percentile rank) and highly abundant proteins (>80 percentile rank)—butanol stress

Gene	Function	RBS score
**CAC0108** ^a^	ABC transporter	12.87
***CAC0091*** ^b^	IlvC	12.38
*CAC1301* ^a^	Hypothetical	9.25

Genes are in italics to indicate their differential proteomic upregulation, while bold represents proteins that were expressed only under butyrate stress and lacked expression under control condition. Bold italics represent non-significant protein expression

^a^5′UTR was not determined due to lack of sufficient data (reads) for CAC0108 and CAC1301

^b^CAC0091 had a 5′UTR of 206 under non-stress condition but was found to be leaderless under butanol stress

Correspondingly, a large number of downregulated proteins, proteins that were expressed under stress and proteins that were expressed only under control conditions, displayed high transcript abundances but had lower levels of proteins. Many of these genes had transcripts with leaderless sequences leading to poor translation.

Finally, the presence of long 5′UTRs for genes that were both transcriptionally and transnationally upregulated were found. One such protein is the Hsp18 containing a 5′UTR of 100 bases, which may play a role in the stability of the transcript and its effective translation under stress conditions. It has a very strong RBS score of 12.87 and was upregulated at the protein level to a maximum of 109- and 111-fold under butyrate and butanol stress, respectively, with similar fold changes (100) reflected at the transcript level. The role of Hsp18 in stress response has been established from previous transcriptional studies on *C. acetobutylicum* [12, 6, 7, 53, 17, 16]. At the protein level, its expression was reported to increase during solventogenesis in comparison to acidogenesis [54, 55] and is also expressed at higher levels in a butanol hyper-tolerant mutant compared to the WT strain [55]. Our analysis here provides insight into a possible role of its long 5′UTR and strong RBS score in achieving these extraordinary high levels of protein upregulation.

## Discussion

There have been nine, genome-scale proteomic studies on *C. acetobutylicum*, most of which were performed using two-dimensional gel electrophoresis combined with mass spectrometry (Table 8) [54–62]. Schaffer et al. (2002) investigated the proteins that were induced during the solventogenic phase of fermentation and identified 86 proteins (52 upregulated and 34 downregulated, fold change ≥2) that had differential expression during solventogenesis [56]. Sullivan and Bennett (2006) analyzed over 200 spots in the WT strain and the Spo0A overexpression strain, for which they analyzed 23 proteins [57]. Among these 23 proteins identified, 22 were also identified in our study, most of which were found to be differentially expressed under stress. Apart from the identification of proteins with differential expression during different metabolic phases of growth and between strains, proteins with more than one spot were also reported, indicating possible post-translational modifications (PTMs; such as phosphorylation, acetylation, or glycosylation). Notably, DnaK, Hsp18, Adc, GroEL, Tpi, Bcd, and Chw16/17 were found to be present in two spots with identical molecular weights but different pI values [57, 56]. Bai and Ji (2012) investigated the phosphoproteome of *C. acetobutylicum* and identified 61 proteins with phosphorylation on the S/T/Y residues, among which 57 proteins were identified in the stress proteome of the current study (56 proteins under butyrate stress and 54 proteins under butanol stress) [58]. Among these 57 phosphoproteins, 31 proteins were found to differentially expressed (FDR 5 %, fold change ≥2) under stress with 17 and 21 proteins differentially expressed under butyrate and butanol stress, respectively.

**Table 8:** Comparison and validation of the proteomic data with earlier reported proteomic work

Work	Brief description	Proteins identified	Proteins found in this work (iTRAQ)	Key findings	Comparison with mRNA expression
Schaffer et al. (2002)	Solventogenesis	130	All 130 proteins have been identified under butanol/butyrate stress	Proteins involved in the solventogenic pathway	Northern analysis (selected few)
Sullivan and Bennett (2006)	Acidogenesis and solventogenesis	23	22 proteins were observed in this study	Proteins expressed during the onset of solventogenesis	With microarray data from Tomas and Alsaker (2004)
Mao et al. (2010)	Cytoplasmic proteins (DSM 1731 and Rh8 tolerant mutant)	564	86 out of the 102 differentially expressed proteins were identified	Proteins that play a role in solvent toxicity tolerance	qRT-PCR of selected differentially expressed protein
Janssen et al. (2010)	Continuous culture of WT in acidogenic and solventogenic phases	178 + 205	178 acidogenic proteins and 205 solventogenic proteins	Proteins that are expressed under different metabolic phases	DNA microarrays
Mao et al. (2011)	Membrane protein DSM 1731 and RH8 tolerant phenotype	341	23 out of the 33 differentially expressed membrane proteins were identified	Membrane proteins that were differentially expressed in the butanol hyper-tolerant mutant	None
Sivagnanam et al. (2011)	CAC proteome under glucose and xylose utilization	717 (glucose) 826 (xylose)	22 of the 23 differentially expressed proteins were identified	Proteins that correspond to differential utilization of carbon sources	None
Bai and Ji (2012)	Phophoproteome of *C. acetobutylicum*	61 phospho-proteins	57/61 phosphor proteins were also identified in our work	Proteins with post translational modifications (PTMs) and their role in stress response	None
Sivagnanam et al. (2012)	Protein interaction network using STRING and CYTOSCAPE	217 proteins were used to construct a PPI network with 1947 interactions	N/A	Construction of PPI network to identify regulatory interactions	None
Jang et al. (2014)	Acidogenesis and solventogenesis. WT, M5, and M5 + pIMP	56	All were also identified by us	Proteins differentially expressed during acidogenic and solventogenic phases	None

Mao et al. (2010) investigated the differential proteome between the WT *C. acetobutylicum* DSM 1731 (presumably the same strain as the type strain ATCC824) and Rh8 hyper-tolerant butanol mutant and identified 102 differentially expressed protein, among which 86 were identified in the current study (42 and 33 were found to be statistically significant, FDR 5 %, under butyrate and butanol stress, respectively) [55].

It was somewhat surprising that a large fraction (23–31 %, by conservative estimation) of the detected proteins in this study displayed opposite differential behavior at the protein versus mRNA level. Disagreements between protein and mRNA levels have been reported in previous studies, all based on microarray data, thus not permitting a detailed interrogation of changes in mRNA composition and structure. These studies included the response of the anaerobe *Desulfovibrio vulgaris* to low oxygen exposure [63], response in *E. coli* to different carbon sources [64], adaptation of *Streptomyces coelicolor* to stationary phase [65], and identification of antibiotic resistance markers in *Staphylococcus aureus* [66]. In some of these studies, they speculated that such disagreements were due to post-transcriptional regulation [67, 68], but no specific mechanisms or explanation were offered except for one, where translational elongation factors were implicated in response to heat shock in a *Synechocystis* sp. [69]. None of these studies has documented mRNA versus protein disagreements as extensive as those documented here. Significantly, here we provide specific mechanistic explanations based on 5′UTR and RBS strength to explain the mRNA-to-protein expression disagreements for a select set of proteins. Furthermore, we document significant and reproducible changes, across several stress conditions, in the expression of proteins in the translation machinery as well as proteins (e.g., the Rho and S10 (CAC3134) proteins) in transcriptional elongation and termination. These data combined with the identification of differential modification of leaderless transcripts provide support for the hypothesis that different ribosome structures are likely utilized to translate select mRNAs under stress, and also that transcriptional elongation and termination are altered under stress to accommodate the cell’s survival program.

Our data provide a rich information basis for more detailed understanding of the complexity of stress response but also several target genes/programs that could be engaged for synthetic purposes, that is, for generating strains with superior tolerance to toxic metabolites.

From the fundamental point of view, an area largely unexplored in Gram^+^ organisms like clostridia is the mechanisms that lead to leaderless transcripts and the physiological role of these select leaderless transcripts. The latter, in fact, remains largely unexplored from the evolutionary point of view. What advantage do leaderless transcripts provide for survival under toxic stress? Perhaps the machinery engaged to translate leaderless transcripts is more robust and selected for operation under stress. What is the physiological reason for which the cells have selected the genes that generate leaderless transcripts under physiological versus stress conditions? What is the mechanism for generation of leaderless transcripts in clostridia and other Gram^+^ organisms? What is the role in this context of the Rho protein, of the toxin-antitoxin system(s), and of the specialized translation initiation factors discussed above?

Two other unexplored areas in this and largely all prokaryotes deserve attention and investigation. The first is the apparent employment, strongly suggested by our data, of different components of the translational machinery (and notably of ribosomal proteins) under normal versus stress conditions. This would suggest that different ribosomes are used under different physiological conditions. Is it possible that some ribosomes are unstable under stress (e.g., solvent stress) conditions while others are not? The second area is strongly suggested by our data differential expression of membrane proteins under stress. While logical in many different ways, the molecular mechanisms by which cells make this selection remains virtually unexplored. In this context, the role of the three components of the SRP (the Ffh protein, the SRP non-coding RNA, and the SRP receptor FtsY) and the differential targeting of membrane proteins by SRP under stress deserve detailed investigations from the fundamental but also synthetic point of view. Could we possibly uncover the membrane proteins that result in more rigid membranes to counteract the chaotropic effect of solvents and acids that diminish or destroy the membrane potential and **Δ**pH? Could we possibly identify the proteins of transporters and related channel proteins that protect the cells from stress through a variety of transport-related mechanisms [4]?

As already stated, our study has identified several genes or programs that could be explored for synthetic applications. Several deserve mentioning here. Engineering cells were based on the SRP system for synthesis of membrane proteins in an exciting possibility. Overexpression and changes in the translational regulation of rubrerythrin should be also explored for enhanced tolerance to acids, oxidative stress, and also likely solvent stress. Understanding the unexplored role of the YacI and YacH proteins of the ClpC operon is another exciting possibility. Also, exploring the role of stress protein in a combinatorial fashion remains largely unexplored as a mechanism for enhanced tolerance. While it is well known that most stress proteins work in ensembles and synergistically, very little of that has been explored and only in *E. coli* ([8, 9]). Is it possible, for example, that the inability of the HSP18 protein to offer enhanced stress tolerance (data not shown), despite the profound upregulation at both the mRNA and protein level, is due to the fact that its partners in action need to also overexpress? Indeed, it is now becoming clear that engineering cells for tolerance is a multicomponent-program goal that requires more sophisticated synthetic biology approaches [8–10, 41, 70].

An interesting possibility is that these new findings can be mapped on and modeled with the recently reconstructed second-generation genome-scale metabolic model (GSM) using the CoreReg method or variations thereof [71], aiming to dissect the dynamics of cell physiology and gene regulation under stress and its subsequent use for metabolic manipulation for design of robust strains.

## Conclusions

This is the first comprehensive system level study to analyze the stress response in *C. acetobutylicum* using multi-omic datasets. Significantly, this is the first reported study to systematically engage proteomic and RNAseq data to focus on genes and programs affected by the phenotypic response where post-transcriptional regulation plays a significant role and provide a mechanistic explanation at the system level for such changes. System level understanding of such post-transcriptional regulation can be effectively employed in synthetic-biology and metabolic-engineering strategies for the development of strains with desirable robust traits.

## Materials and methods

### Bacterial strains and stress cultures

Three biological replicates of *C. acetobutylicum* ATCC824 were grown anaerobically in a pH-controlled (pH >5) batch fermentation in a 4 L New Brunswick BioFlo 310 bioreactor as described earlier [15, 16]. The cultures were stressed with butyrate (low - 30 mM, med - 40 mM, high - 50 mM) and butanol (low - 30 mM, med - 60 mM, high - 90 mM) at mid-exponential growth phase at an OD of 1.0. A non-stressed culture was used as the control. Samples for RNA and protein extractions were obtained at regular intervals of 0, 15, 30, 45, 60, and 75 min post stress. The proteomic analyses was performed at 15, 45, and 75 min.

#### Transcriptomic datasets for differential expression analysis

Transcriptomic analyses were performed using microarrays (GEO datasets GSE48031 and GSE48039) at all six time points [16] and RNAseq [15] (GEO dataset GSE48349) at four time points (15, 30, 60, and 75 min). The data were normalized and analyzed for differential expression using DESeq [72] as described previously [15]. For both transcriptomic techniques, validation was performed using qRT-PCR as reported earlier [15, 16].

#### Strand-specific RNAseq analysis and determination of TSS, UTRs, and operon structures

Strand-specific RNAseq was performed using libraries from non-stress control, high butanol, and high butyrate stress conditions at two time points, 75 and 270 min post stress. Following RNA isolation using Qiagen miRNeasy kit and rRNA removal using Ambion MICROBExpress kit, the RNA were also subjected to a Terminator™ 5′-phosphate dependent exonuclease (TEX) treatment for the enrichment of 5′ end of the RNA containing TSS. The libraries were prepared using ScriptSeq V2 (Epicentre, Illumina) and sequenced using paired-end (75 cycles) Illumina HiSeq 2500 at Delaware Biotechnology Institute. Following the trimming [73] of adapters, the data was analyzed using the Rockhopper software [74] for alignment of reads to the reference genome, data normalization, differential expression, TSS prediction, and operon organization. The data has been submitted to NCBI’s sequence read archives under the BioProject PRJNA273734 containing 30 BioSamples (SAMN03295242-SAMN03295271).

#### Protein extraction, digestion, and iTRAQ labeling

*Clostridium acetobutylicum* wild-type (WT) cells were cultured under low, medium, and high levels of butanol (BuOH) or butyrate (BA) stress or no stress. For proteomic analysis, cell pellets were resuspended in 100-mL lysis buffer containing 0.01 % SDS and 0.5-M triethylammonium bicarbonate (TEAB) buffer (pH 8.5) and sonicated with 1 % (w/v) calcium carbonate as previously reported [75]. Cell lysates were centrifuged at 20,000 *g* at 4 °C for 10 min, and concentration of total protein in the supernatant was determined by Bradford assay (Thermo Fisher Scientific Inc., Rockford, IL, USA). Samples with 100-μg protein from each culture condition were reduced, alkylated, digested, and cleaned up as previously reported [75]. The digests were concentrated to 30 μL then labeled with iTRAQ 4-plex labels (AB Sciex, Foster City, CA, USA) per manufacturer’s instruction according to the labeling scheme listed in Additional file 1: Table S1.

#### Two-dimensional liquid chromatography (2D-LC) and mass spectrometry (MS) data acquisition

Labeled samples were combined and separated by high pH reverse phase LC (RPLC) followed by second dimension RPLC as described [75]. The eluate was introduced to an in-line QTrap 4000 (AB Sciex) through a nanoSpray II source (AB Sciex) using an uncoated fused-silica Pico tip (New Objective, Woburn, MA, USA). MS/MS data were acquired as described [75].

#### Protein sequence database search and data analysis

For protein identification and quantification, raw MS/MS data were submitted to Paragon in ProteinPilot (version 3, AB Sciex) and searched against a local *CAC* sequence database (a concatenation of NCBI references NC_003030.1 and NC_001988). Search parameters were the same as previously specified [75]. Bias correction and background correction were performed through ProteinPilot. Protein identifications were based on 95 % confidence or above, and only proteins with at least one peptide with 95 % confidence were included in the quantified protein list. For evaluation of the protein identification false discovery rate (FDR), the MS/MS data were submitted to a decoy database and FDR was calculated from the ratio of the number of hits from the decoy database to the number of hits from normal and decoy database (Additional file 2: Proteomic data).

Protein expression levels under BuOH or BA stress were compared to the controls under no stress with the same reference using significance analysis of microarrays (SAM analysis [76]) with MeV v4.8 [77] as reported [75]. The delta values were set for an FDR of 5 % as cutoff.

## Additional files

Additional file 1:
**Supplemental information.** Additional figures and descriptions on integrative proteomic and transcriptomic analyses. Additional file 2:
**Proteomic data.** *Clostridium acetobutylicum* proteomic data for butanol and butyrate stress using iTRAQ labeling.

## Abbreviations

5′UTR: 5′ untranslated region
ABE: acetone, butanol, and ethanol
COG: Cluster of Orthologous Groups
HSP: heat shock protein
iTRAQ: isobaric tag for relative and absolute quantitation
RBS: ribosomal binding site
SAM: significant analysis of microarrays
SRP: signal recognition particle
TSS: transcriptional start site
