# Supplementary material for: Complex and extensive post-transcriptional regulation revealed by integrative proteomic and transcriptomic analysis of metabolite stress response in Clostridium acetobutylicum
Source: Biotechnol Biofuels. 2015 Jun 10;8:81. doi: 10.1186/s13068-015-0260-9 (PMC4533764; doi:10.1186/s13068-015-0260-9)
Supplement: Additional file 1: — Supplemental information. Additional figures and descriptions on integrative proteomic and transcriptomic analyses. [file 13068_2015_260_MOESM1_ESM.pdf]

Supplementary Material

Complex and extensive post-transcriptional regulation revealed by integrative proteomic & transcriptomic analysis of metabolite stress response in *Clostridium acetobutylicum*

Table S1. iTRAQ labeling scheme

Stress type	Stress level	iTRAQ tags			
		114	115	116	117
BuOH	High	Pooled[a]	15 min	45 min	75 min
	Medium	75 min	Pooled[a]	15 min	45 min
	Low	45 min	75 min	Pooled[a]	15 min
	No	15 min	45 min	75 min	Pooled[a]
BA	High	Pooled[b]	75 min	45 min	15 min
	Medium	75 min	Pooled[b]	15 min	45 min
	Low	45 min	15 min	Pooled[b]	75 min
	No	15 min	45 min	75 min	Pooled[b]

Reference channels pooled[a] and pooled[b] are created for comparing different sets of iTRAQ data by mixing equal amounts of samples with the same reference.

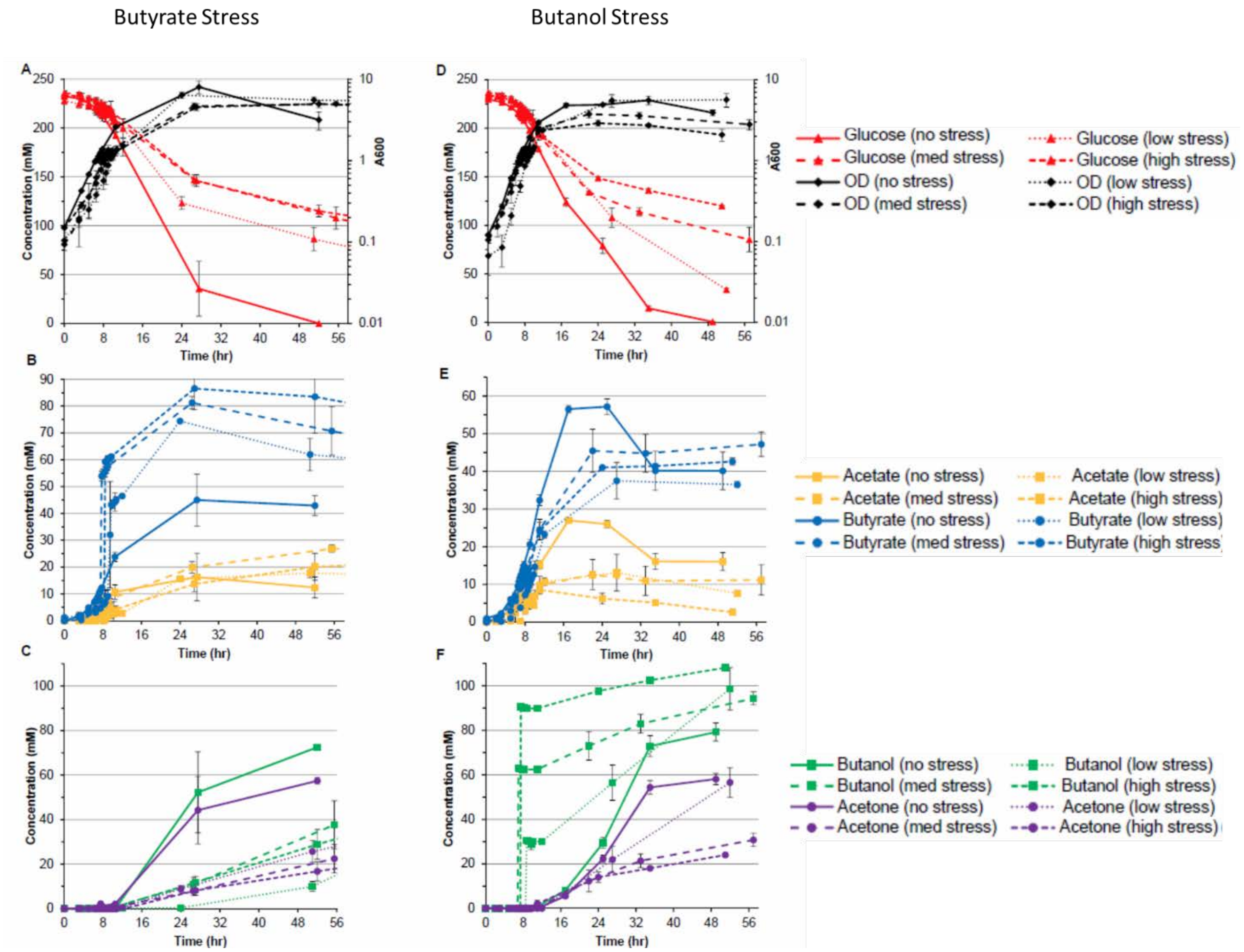

Supplementary Figure S1. Growth and metabolite profile of C. acetobutylicum under stress. Butyrate stress (A, B, C) and Butanol stress (D, E, F).

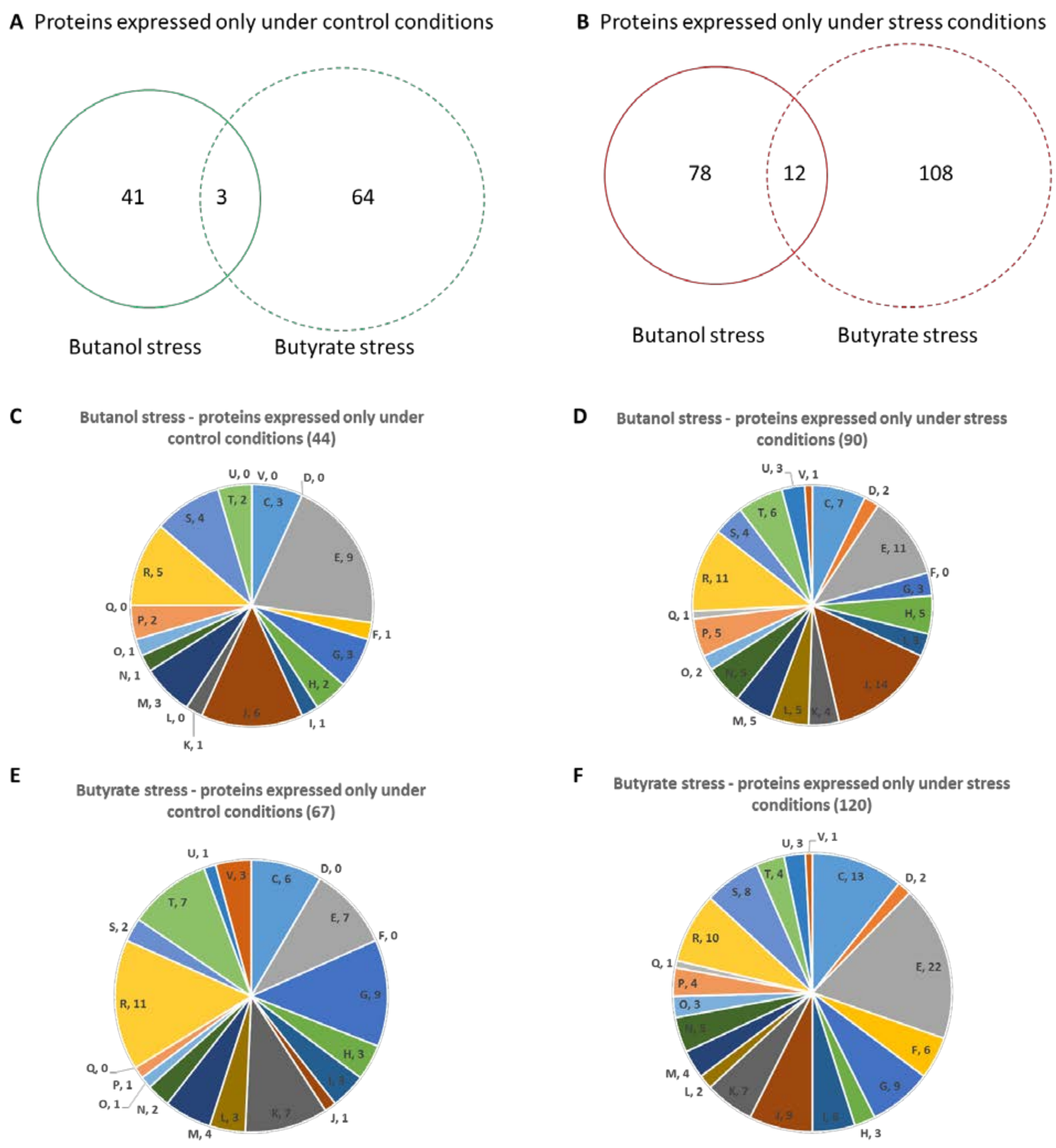

Supplementary Figure S2. (A) Comparison of proteins expressed only under control condition between butanol and butyrate stress. (B) Comparison of proteins expressed only stress control condition between butanol and butyrate stress. COG distribution under butanol stress (C) proteins expressed only under control conditions, (D) proteins expressed only stress control

conditions. COG distribution under butyrate stress (C) proteins expressed only under control conditions, (D) proteins expressed only stress control conditions.

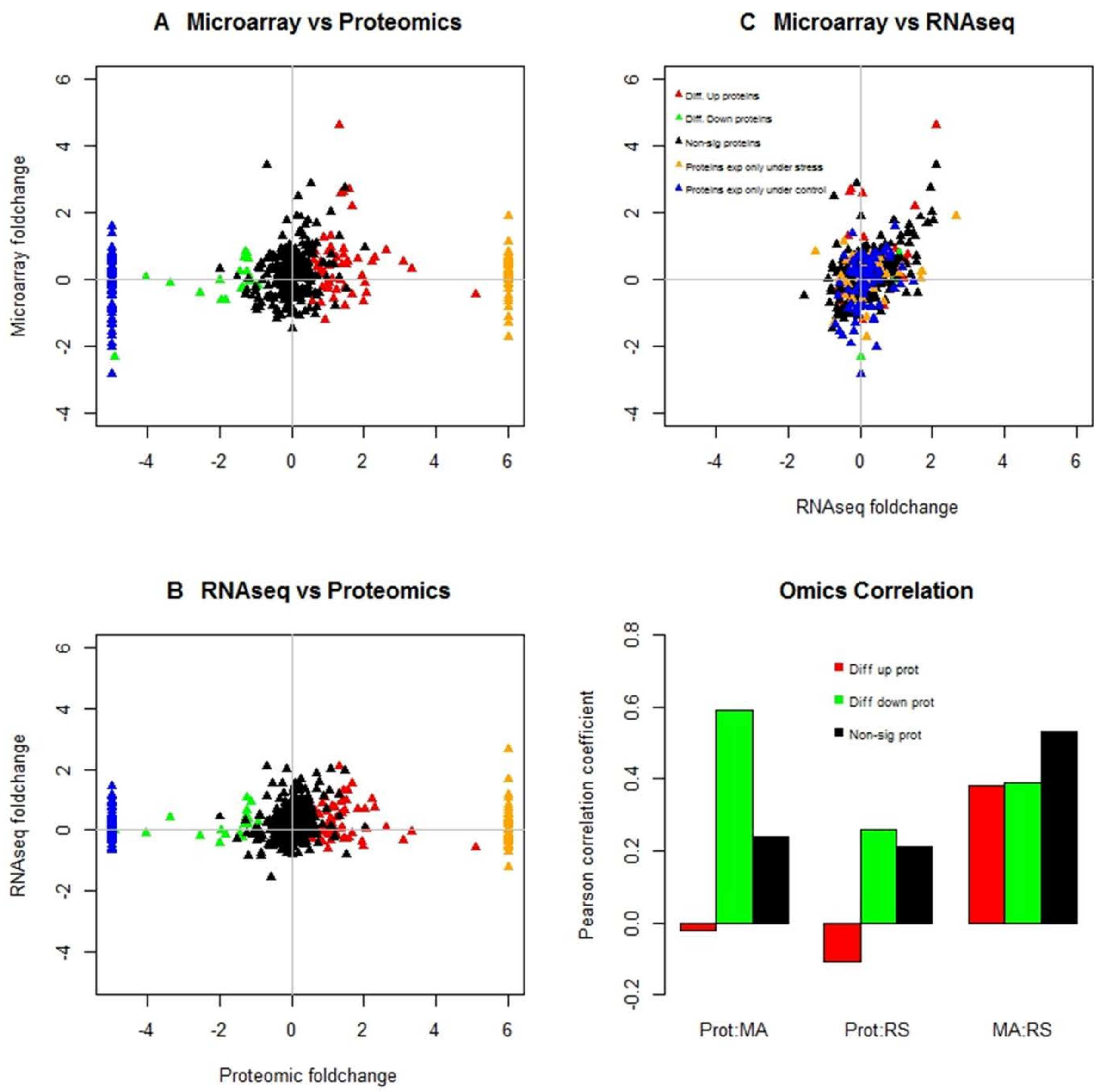

Supplementary Figure S3. Comparison and correlation between proteomic and transcriptomic data under low butyrate stress. A) Microarray vs proteomic comparison. B) RNAseq vs proteomic comparison. C) Microarray vs RNAseq comparison. D) Pearson correlation. All significant expression are with respect to proteomic data only. Red – differentially upregulated proteins; Green – differentially downregulated proteins; Black – non-

significant proteins; Blue – proteins expressed only under non-stress control; Orange – proteins expressed only under stress.

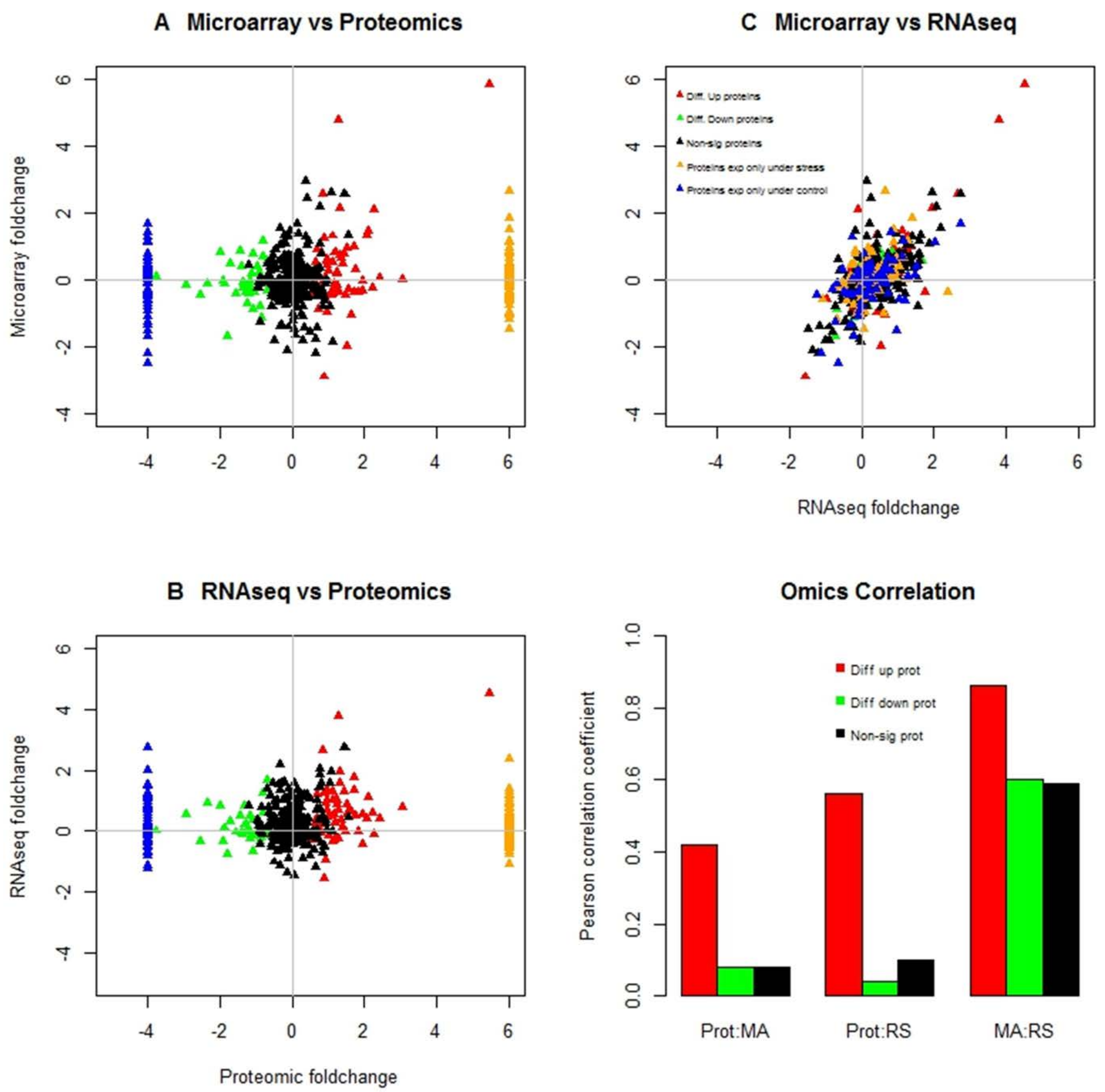

Supplementary Figure S4. Comparison and correlation between proteomic and transcriptomic data under medium butyrate stress. A) Microarray vs proteomic comparison. B) RNAseq vs proteomic comparison. C) Microarray vs RNAseq comparison. D) Pearson correlation. All significant expression are with respect to proteomic data only. Red – differentially upregulated proteins; Green – differentially downregulated proteins; Black – non-

significant proteins; Blue – proteins expressed only under non-stress control; Orange – proteins expressed only under stress.

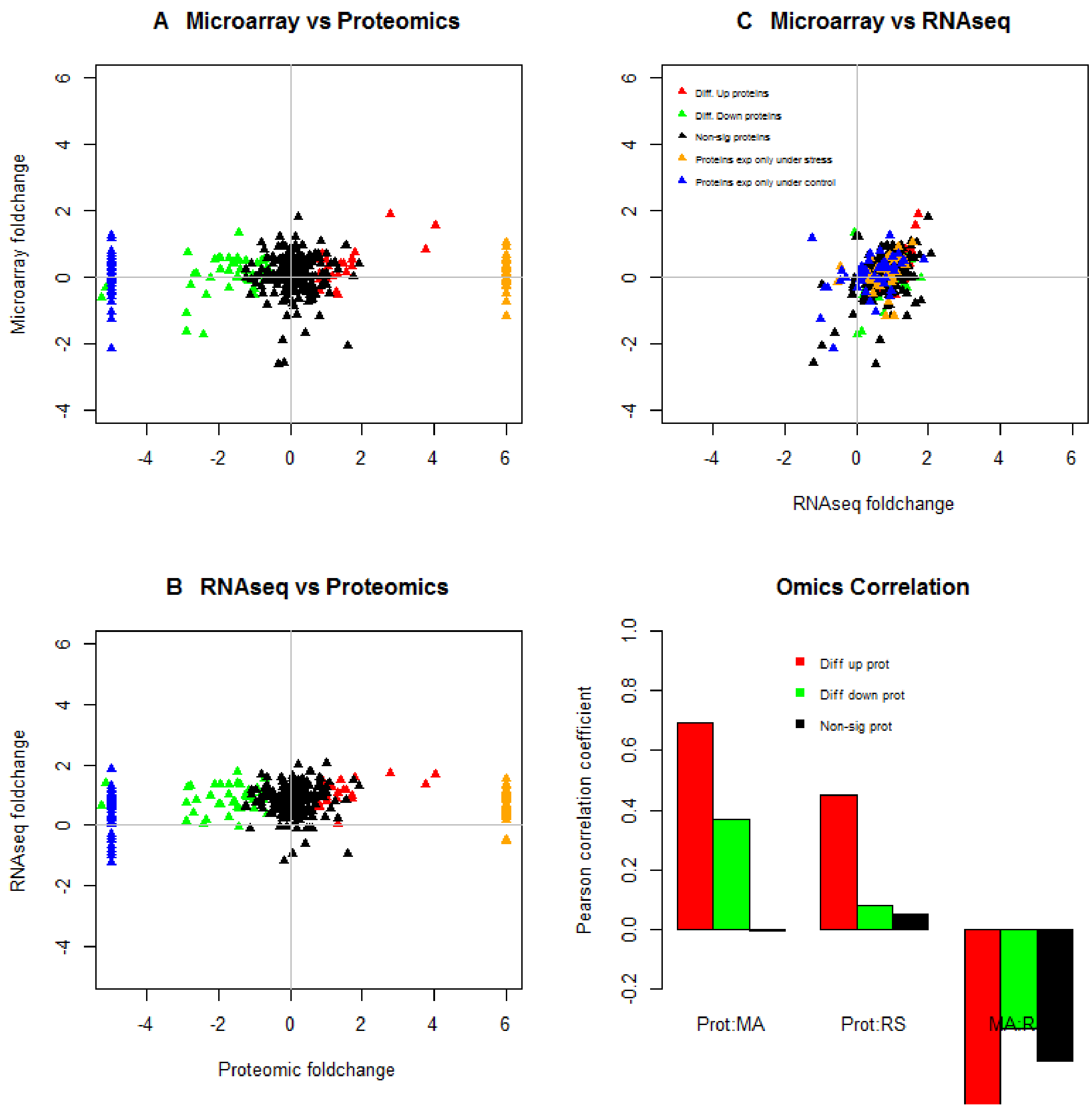

Supplementary Figure S5. Comparison and correlation between proteomic and transcriptomic data under low butanol stress. A) Microarray vs proteomic comparison. B) RNAseq vs proteomic comparison. C) Microarray vs RNAseq comparison. D) Pearson correlation. All significant expression are with respect to proteomic data only. Red – differentially upregulated proteins; Green – differentially downregulated proteins; Black – non-

significant proteins; Blue – proteins expressed only under non-stress control; Orange – proteins expressed only under stress.

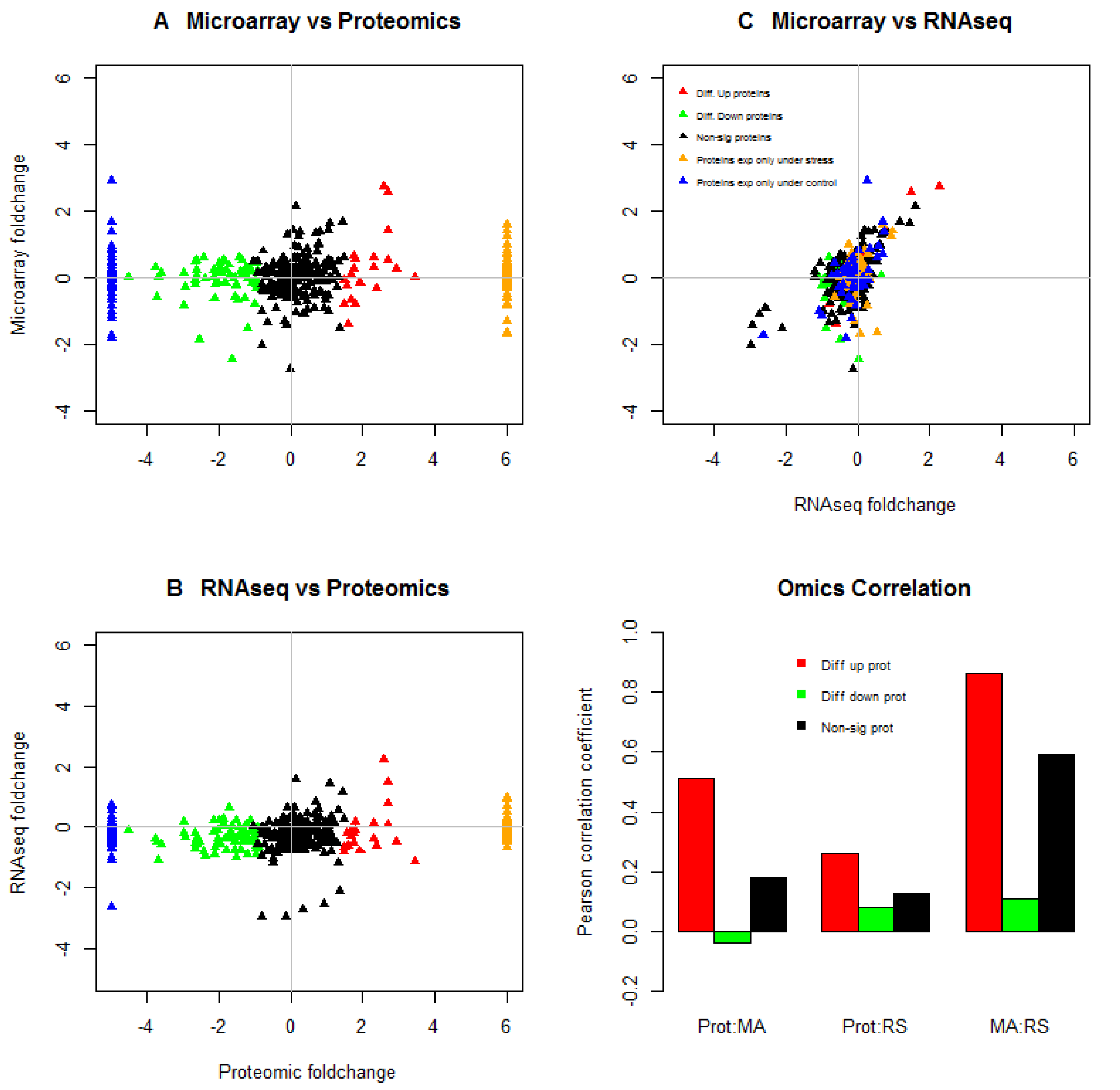

Supplementary Figure S6. Comparison and correlation between proteomic and transcriptomic data under medium butanol stress. A) Microarray vs proteomic comparison. B) RNAseq vs proteomic comparison. C) Microarray vs RNAseq comparison. D) Pearson correlation. All significant expression are with respect to proteomic data only. Red – differentially upregulated proteins; Green – differentially downregulated proteins; Black – non-

significant proteins; Blue – proteins expressed only under non-stress control; Orange – proteins expressed only under stress.

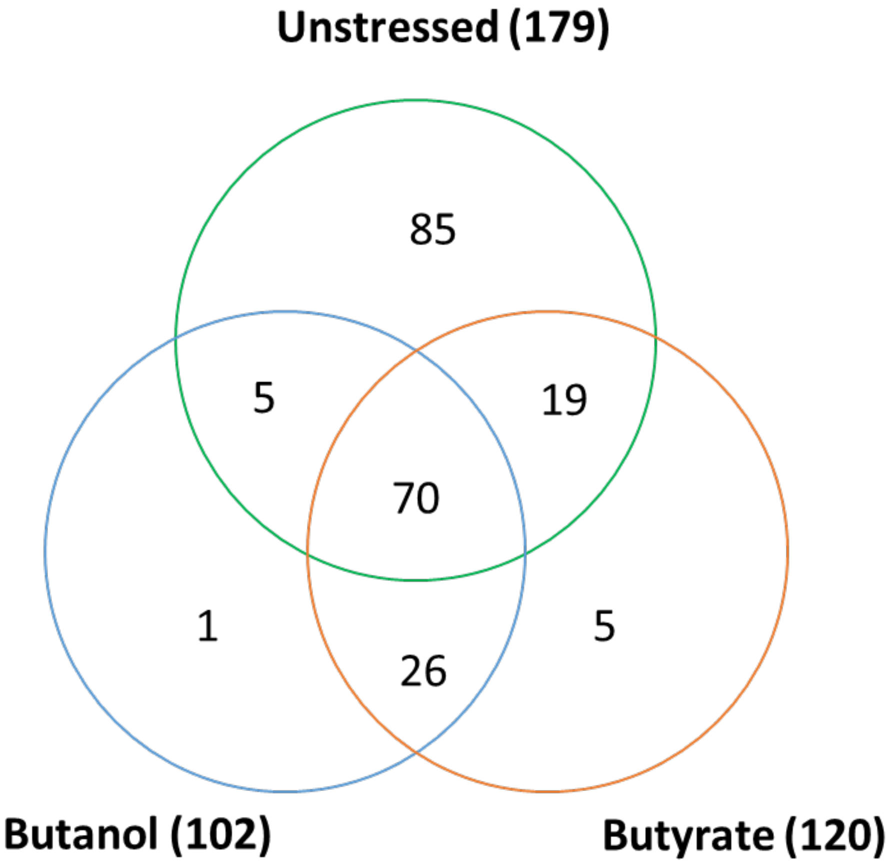

Supplementary Figure S7. Comparison of proteins with leaderless transcripts as identified using strand-specific sequencing.

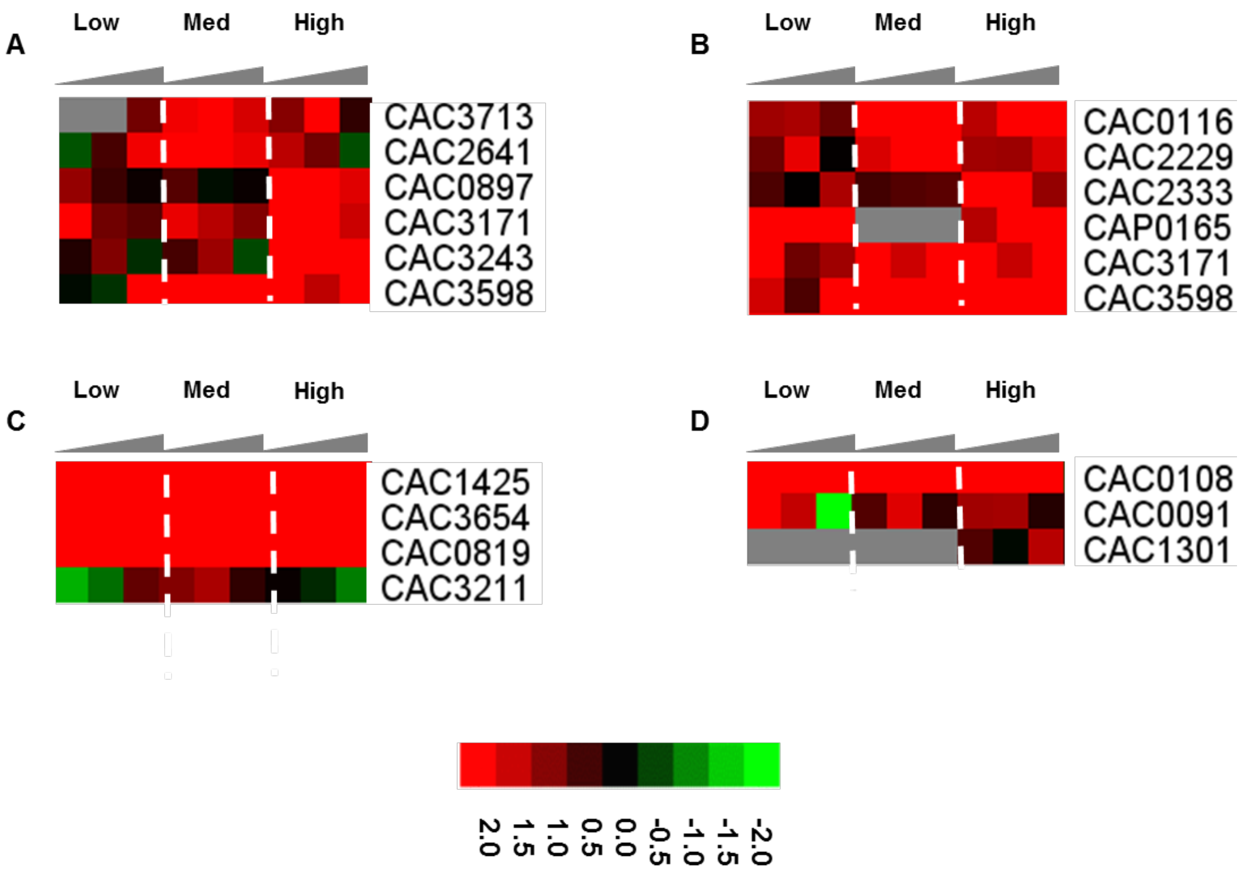

Supplementary Figure S8. Heat map of proteins in Table 3.

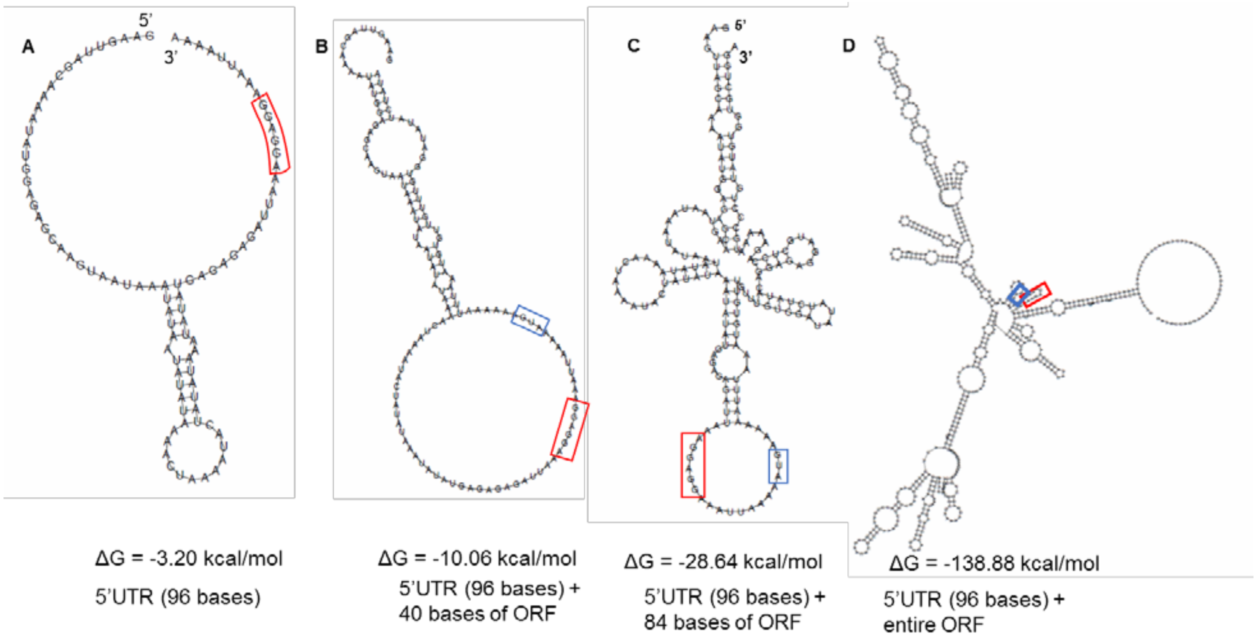

Supplementary Figure S9. RNAfold of rubrerythrin (CAC3598) transcript. (A) 5' UTR; (B) 5'UTR with 40 bases of ORF; (C) 5'UTR with 84 bases of ORF and (D) 5'UTR and the entire ORF.

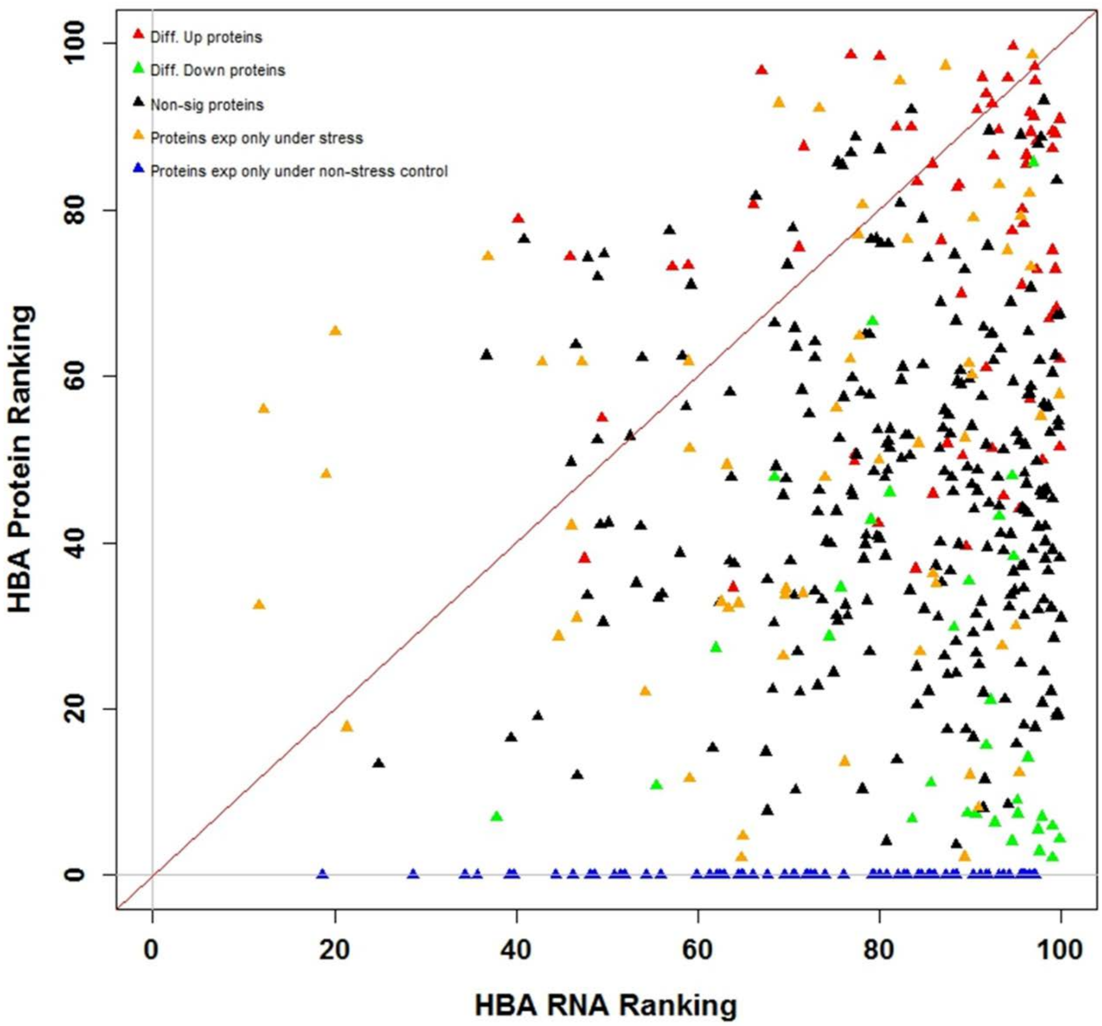

Supplementary Figure S10. Comparison of the RNAseq transcript abundance percentile ranking for proteins detected using iTRAQ 4-plex under high butyrate stress (HBA). X-axis – average percentile transcript abundance ranking under stress and y-axis represents average percentile transcript abundance ranking under non-stress control. Red – differentially upregulated proteins; Green – differentially downregulated proteins; Black – non-significant proteins; Blue – proteins expressed only under non-stress control; Orange – proteins expressed only under stress.

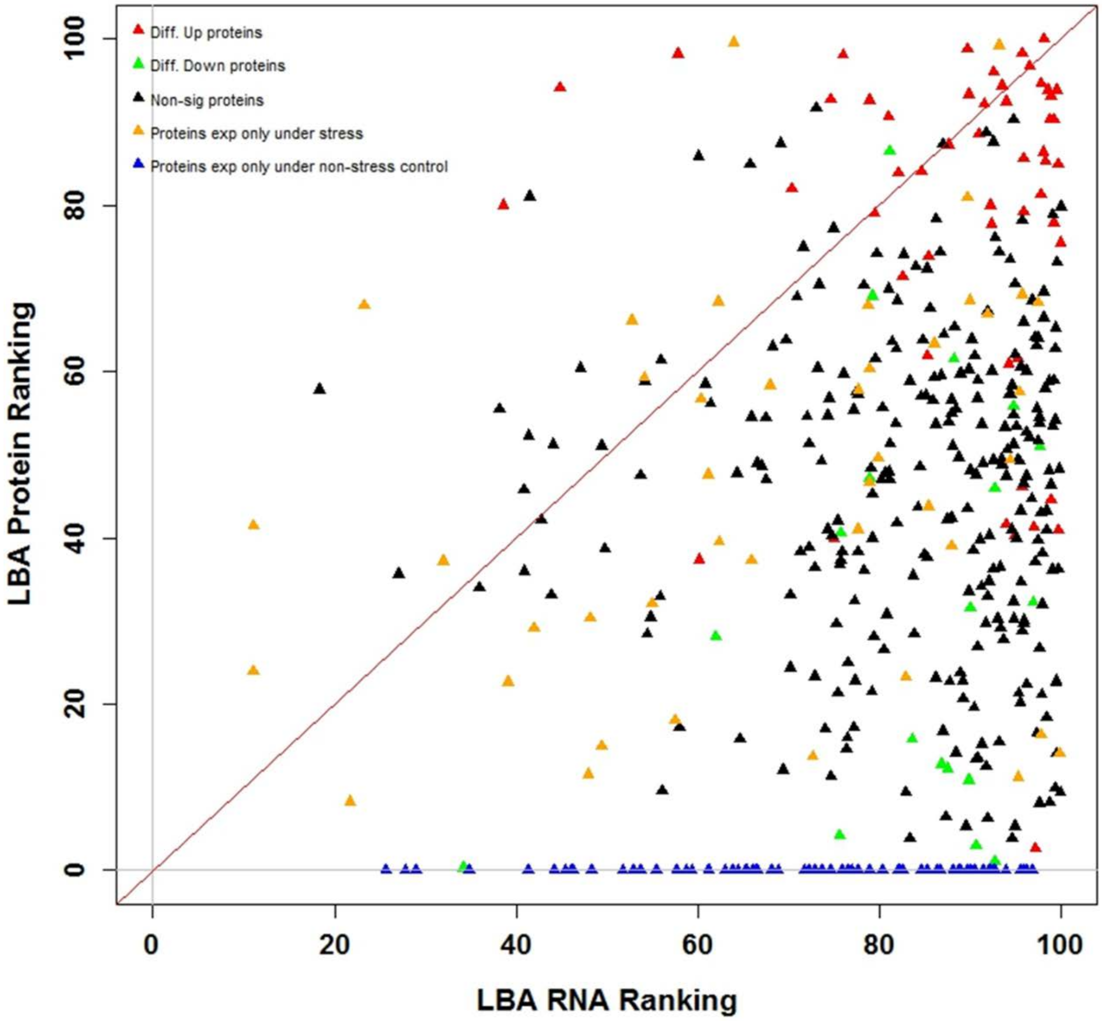

Supplementary Figure S11. Comparison of the RNAseq transcript abundance percentile ranking for proteins detected using iTRAQ 4-plex under low butyrate stress (LBA). X-axis – average percentile transcript abundance ranking under stress and y-axis represents average percentile transcript abundance ranking under non-stress control. Red – differentially upregulated proteins; Green – differentially downregulated proteins; Black – non-significant proteins; Blue – proteins expressed only under non-stress control; Orange – proteins expressed only under stress.

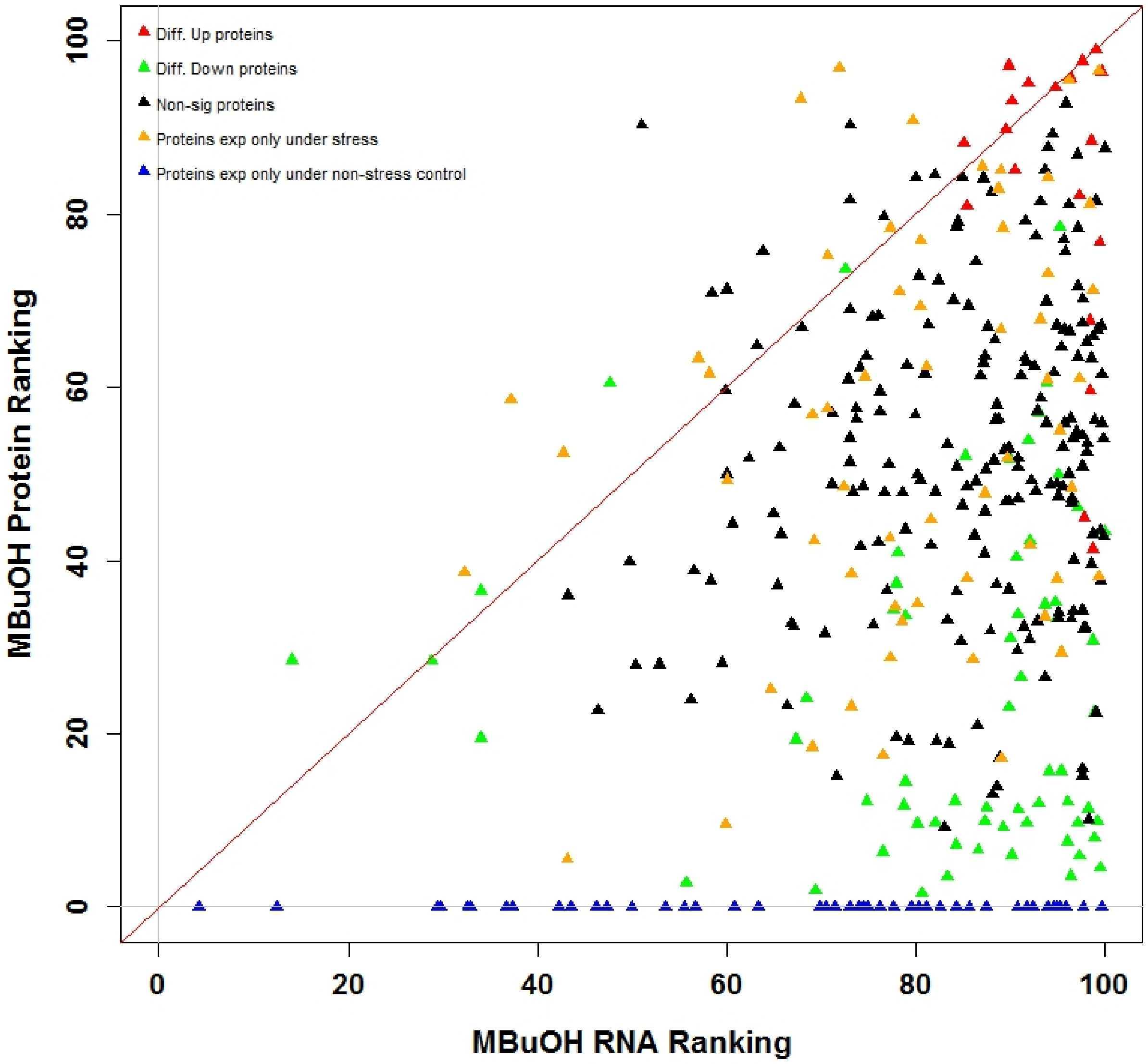

Supplementary Figure S12. Comparison of the RNAseq transcript abundance percentile ranking for proteins detected using iTRAQ 4-plex under medium butanol stress (MBuOH). X-axis – average percentile transcript abundance ranking under stress and y-axis represents average percentile transcript abundance ranking under non-stress control. Red – differentially upregulated proteins; Green – differentially downregulated proteins; Black – non-significant

proteins; Blue – proteins expressed only under non-stress control; Orange – proteins expressed only under stress.

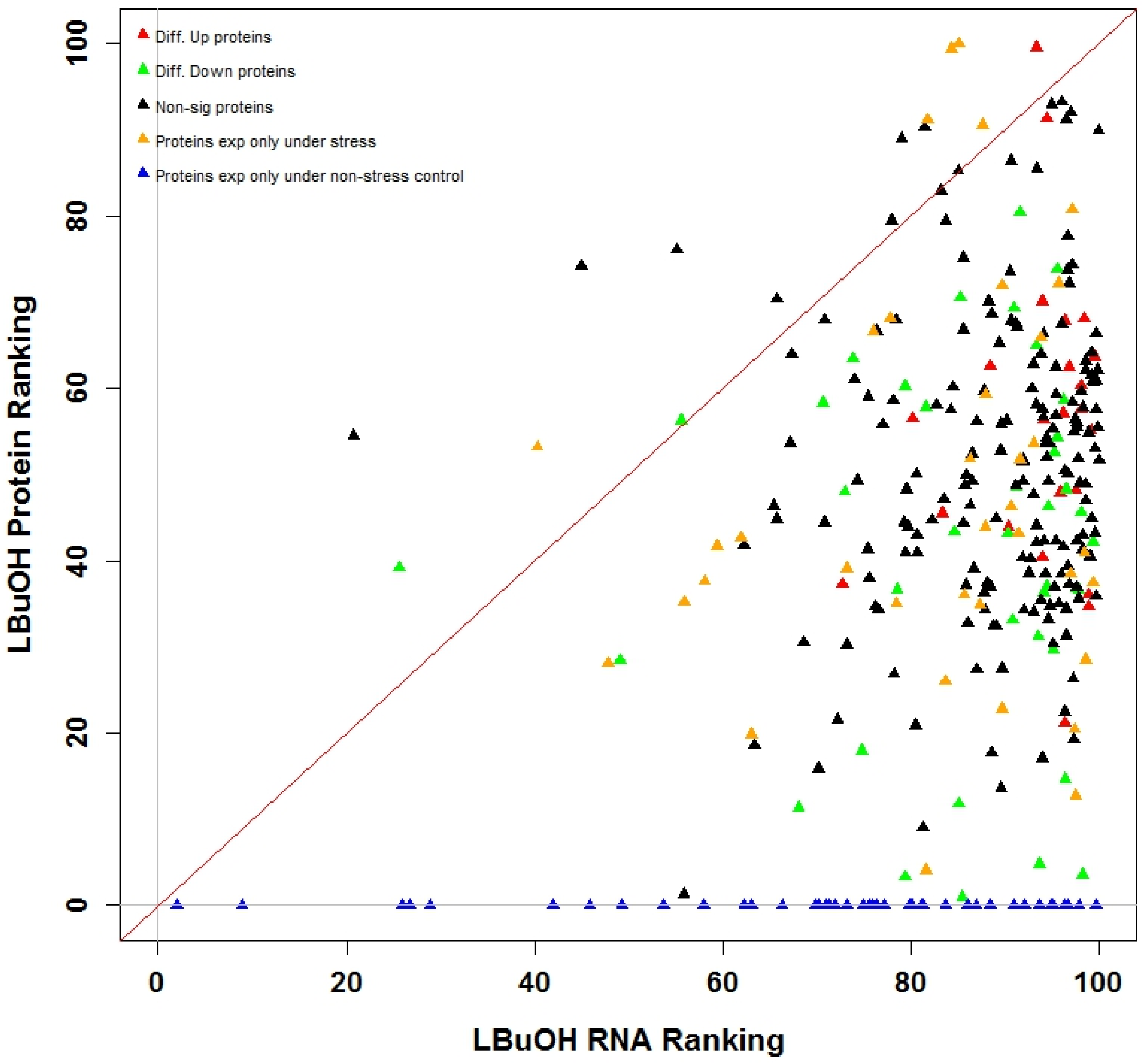

Supplementary Figure S13. Comparison of the RNAseq transcript abundance percentile ranking for proteins detected using iTRAQ 4-plex under low butanol stress(LBuOH). X-axis – average percentile transcript abundance ranking under stress and y-axis represents average percentile transcript abundance ranking under non-stress control. Red – differentially upregulated proteins; Green – differentially downregulated proteins; Black – non-significant

proteins; Blue – proteins expressed only under non-stress control; Orange – proteins expressed only under stress.

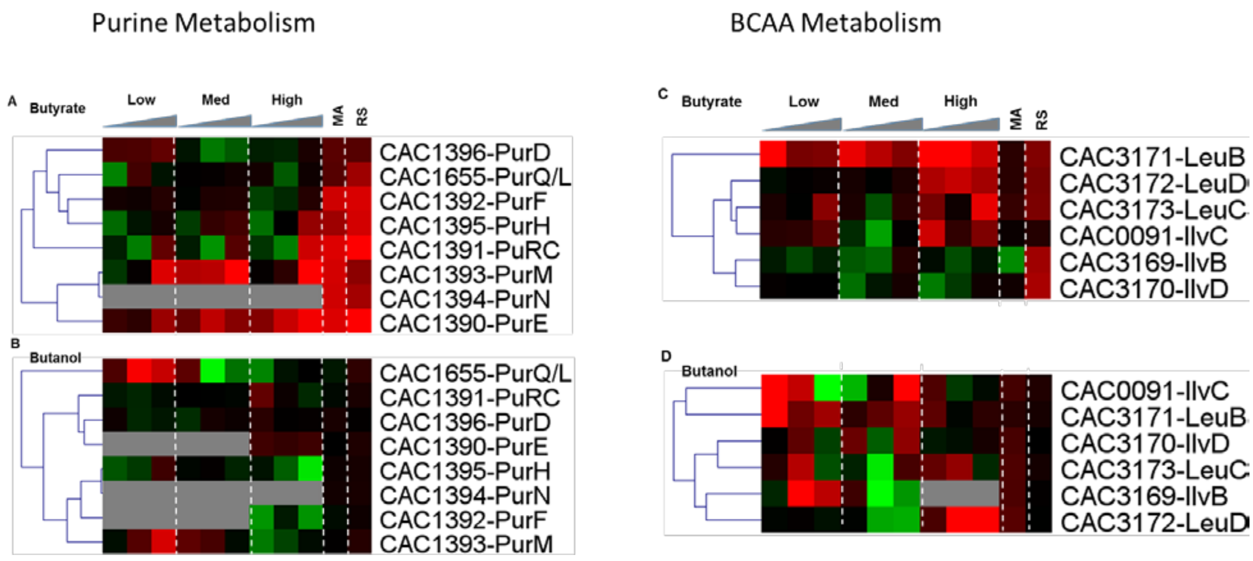

Supplementary Figure S14: Comparison of the proteomic and transcriptomic expression of Purine metabolism (A) butyrate stress (B) butanol stress and branched chain amino acid (BCAA) metabolism (C) butyrate stress (D) butanol stress. The microarray (MA) and RNAseq (RS) values are displayed as average fold change across all time points.

Under butyrate stress, the genes involved in the purine metabolism (CAC1390-CAC1396 and CAC1655) were found to be upregulated at the mRNA level but were found to be downregulated at the protein level except for PurM (CAC1393) and PurE (CAC1390) which were upregulated at the protein level (Supplementary Figure S1a). The downregulation of the purine metabolism genes at the protein level shows possible post-transcriptional regulation as mentioned earlier. On the other hand, under butanol stress, the purine metabolism genes had a non-significant expression at mRNA level and at protein level (Supplementary Figure S1b), suggesting the levels of mRNA and protein for purine metabolism was not affected under butanol stress.

Similarly the branched chain amino acid synthesis pathway (CAC3169-CAC3173 & CAC0091) responsible for the synthesis of isoleucine, leucine and valine was found to be upregulated at the

protein under butyrate stress even though the mRNA were not differentially upregulated (Supplementary Figure S11c). This again points to post-transcriptional regulation at the translational level. Under butanol stress, the ILV synthesis pathway is upregulated at low butanol stress (Supplementary Figure S11d). It has been postulated that the increase in the synthesis of ILV is a response of the cells to modify the membrane (homeoviscous response), as part of the cellular response to butanol stress. Furthermore the flux in the ILV synthesis pathway was reported to be upregulated under stress [52]. Thus, the proteomic data for the branched chain amino acids confirms its role in stress responsive metabolism from previous studies and metabolomics data.
